# Supplementary material for: Tracking longitudinal population dynamics of single neuronal calcium signal using SCOUT
Source: Cell Rep Methods. 2022 Apr 29;2(5):100207. doi: 10.1016/j.crmeth.2022.100207 (PMC9142684; doi:10.1016/j.crmeth.2022.100207)
Supplement: Document S1. Figures S1–S6 and Table S1 [file mmc1.pdf]

Cell Reports Methods, Volume *2*

Supplemental information

Tracking longitudinal population dynamics of single neuronal calcium signal using SCOUT

Kevin G. Johnston, Steven F. Grieco, Hai Zhang, Suoqin Jin, Xiangmin Xu, and Qing Nie

Supplemental Figure 1

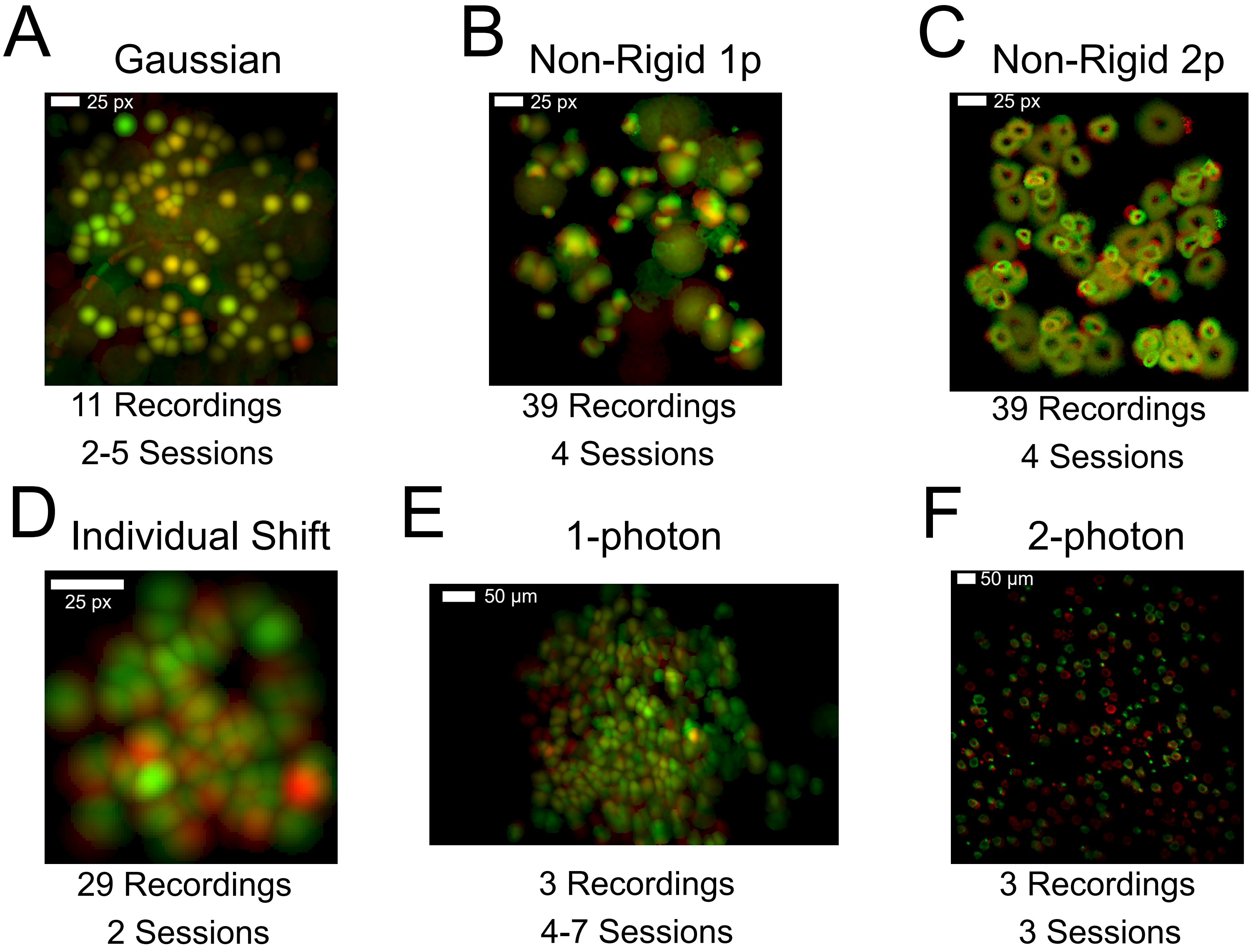

Supplemental Figure 1: **Overview of tested data, Related to Figures 3-5**. Neuron extraction from two sessions from each dataset, overlaid to visualize the identification of neurons across each session, as well as the number of recordings and sessions per recording in each dataset. Gaussian (**A**), Non-Rigid 1p (**B**), Non-Rigid 2p (**C**), and Individual Shift (**D**) datasets are simulated, while 1-photon (**E**) and 2-photon (**F**) datasets are *in vivo* recordings. Simulated datasets exhibit different features and difficulties for cell identification across sessions, such as noise (particularly in the Non-Rigid 1p dataset), cell body transformations (Non-Rigid 1p and Non-rigid 2p) and neuron translations (Individual Shift) represent common issues with *in vivo* recordings. Similar effects can be viewed in the *in vivo* recording representations.

Supplemental Figure 2

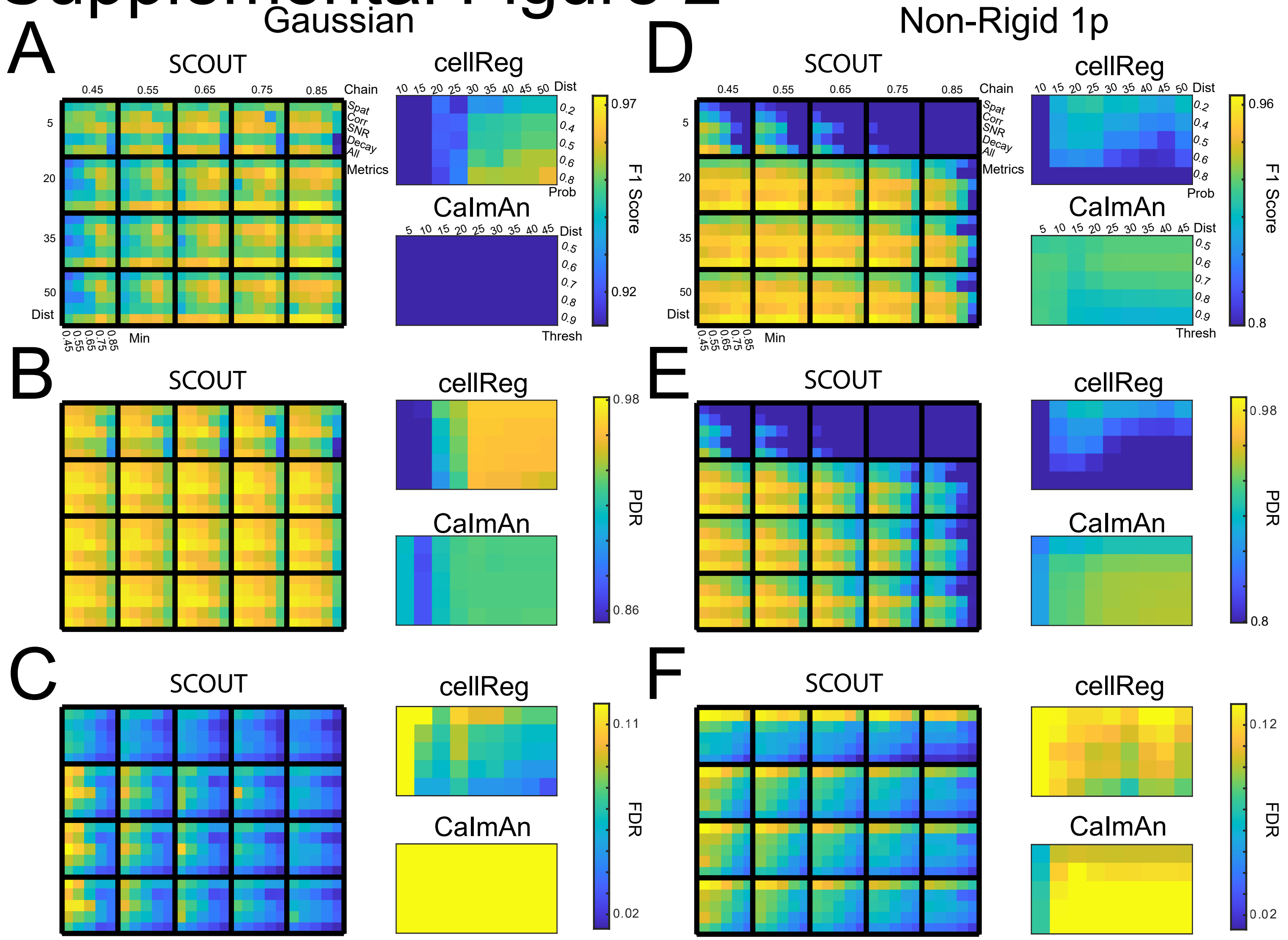

Supplemental Figure 2: **Comparison of F1, PDR and FDR scores on the Gaussian and Non-Rigid 1p datasets, Related to Figure 3. A-E**: Each heatmap depicts a different metric for determining cell tracking quality: PDR the percentage of tracked neurons through all recordings of those present in each recording; FDR the percentage of tracked neurons containing at least one identification error; F1 Score defined as $F1 = 2 * PDR * (1 - FDR)/(1 - FDR + PDR)$. Parameters are labeled in **A(D)** and extend to **B-C(E-F)**. Parameters for SCOUT include *max_dist* (labeled Dist), *min_prob* (labeled min), and *chain_prob* (labeled chain). The vertical change in each box corresponds to which metrics were used in the computation (spatial only, correlation, SNR, decay, or all metrics), the horizontal change in each box corresponds to variation in the *min_prob* parameter. Vertical change across boxes corresponds to variation in the *max_dist* parameter, and horizontal changes across boxes correspond to the *chain_prob* parameter. CaImAn and cellReg have a common maximum centroid distance (Dist) parameter, as well as threshold parameters (Prob, Thresh) governing acceptance levels for tracked cells. Results for Gaussian dataset on left (**A-C**), results for Non-Rigid 1p dataset on right (**D-F**).

Supplemental Figure 3

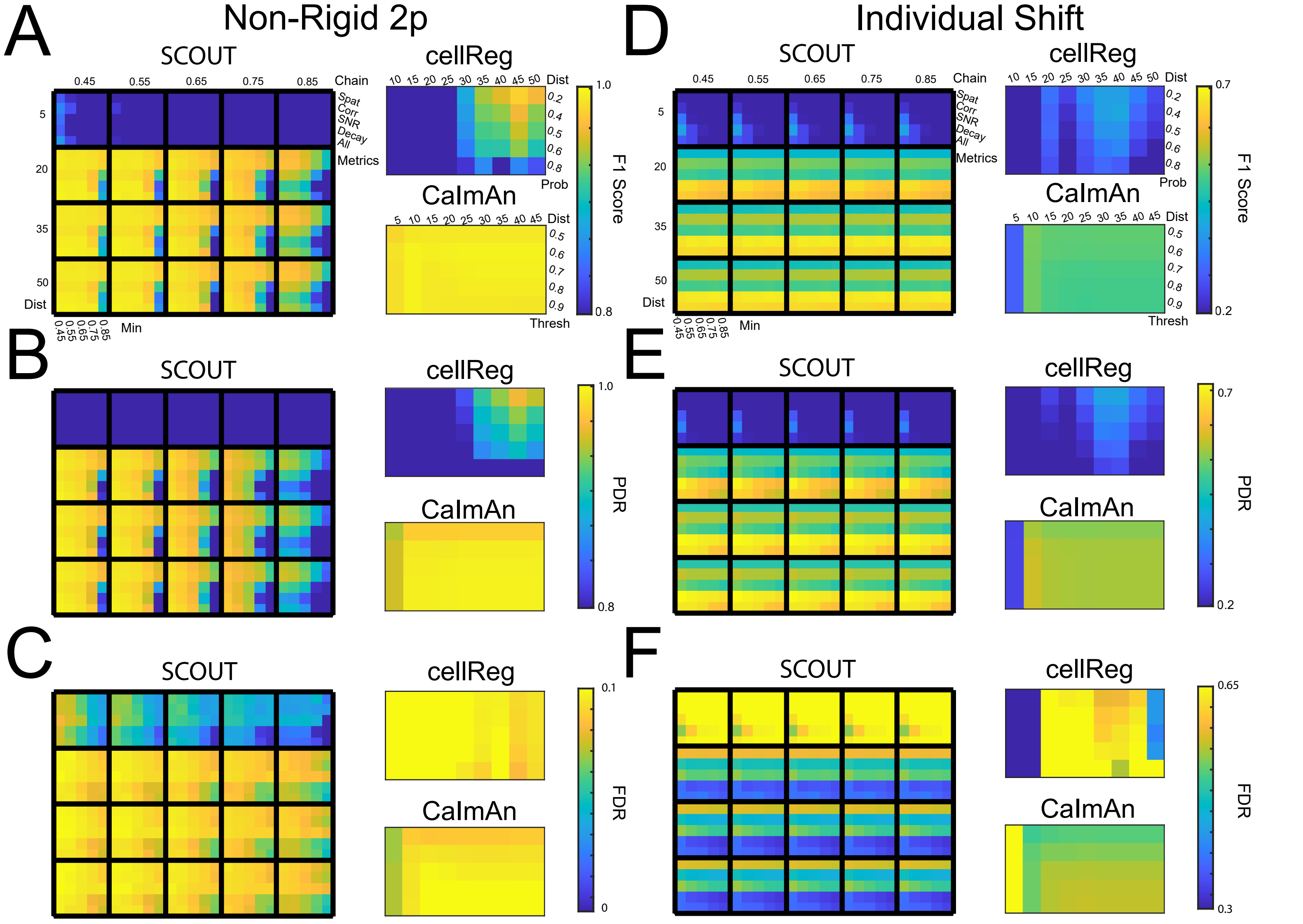

Supplemental Figure 3: **Comparison of F1, PDR, and FDR scores on the Non-Rigid 2p and Individual Shift datasets, Related to Figure 3. A-C**: F1 scores (**A**), PDR (**B**), and FDR (**C**) for SCOUT, cellReg, and CaImAn across a variety of parameters for the Non-Rigid 1p dataset. Parameter labels (**A**) extend to **B-C**. **D-F**: F1 scores (**D**), PDR (**E**), and FDR (**F**) for SCOUT, cellReg, and CaImAn across a variety of parameters for the Non-Rigid 1p dataset. Parameter labels (**D**) extend to **E-F**. Results for the Non-Rigid 2p dataset on the left (**A-C**), results for the Individual Shift dataset on the right (**D-F**).

Supplemental Figure 4

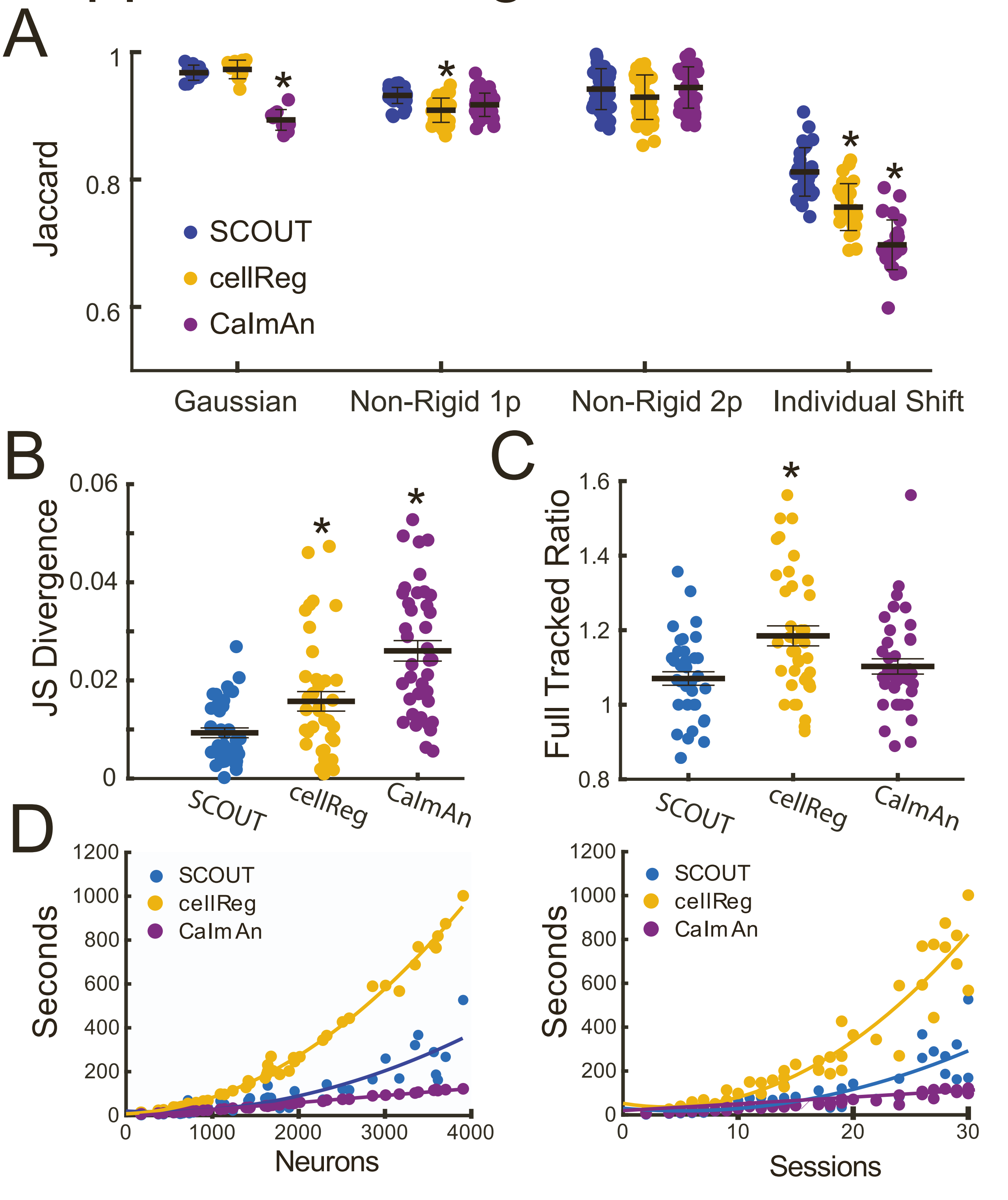

Supplemental Figure 4: Supplemental statistical comparisons of methods, Related to Figure 3. A: Maximal Jaccard similarity (y-axis) on modified versions of the simulated datasets (x-axis), in which 30% of neurons were removed from analysis prior to cell tracking. **B:** JS divergence (y-axis) between cluster size distributions of inferred cell registers for each method (x-axis), on the Non-Rigid 1p dataset after removal of 30% of neurons, based on parameters producing highest F1 scores. **C:** Full tracking ratios (y-axis, the percentage of available neurons tracked through all sessions) on the modified Non-Rigid 1p dataset based on parameters producing highest F1 scores. **D:** Algorithm runtime comparison between SCOUT, cellReg and CaImAn. Testing was performed on the Non-Rigid 1p dataset. We performed 50 tests in the following manner: 1) a random recording was identified; 2) recording sessions were duplicated to construct up to 30 sessions; 3) SCOUT, cellReg and CaImAn were run with global session registration disabled, and without the JS metric (SCOUT only) to provide the most unbiased results. Tests were run with 24 cores on a 2.2 Ghz CPU. Results are shown based on the seconds required to complete cell tracking (y-axis), and the total number of neurons (left) or sessions (right) in the recording. SCOUT and cellReg results were fit with a quadratic curve, and CaImAn with a linear curve. For panels **A-C**, statistical results denote pairwise comparison with SCOUT and are labeled with black asterisks (ANOVA, Bonferroni multiple comparisons). Black bars indicate mean ± SE.

Supplemental Figure 5

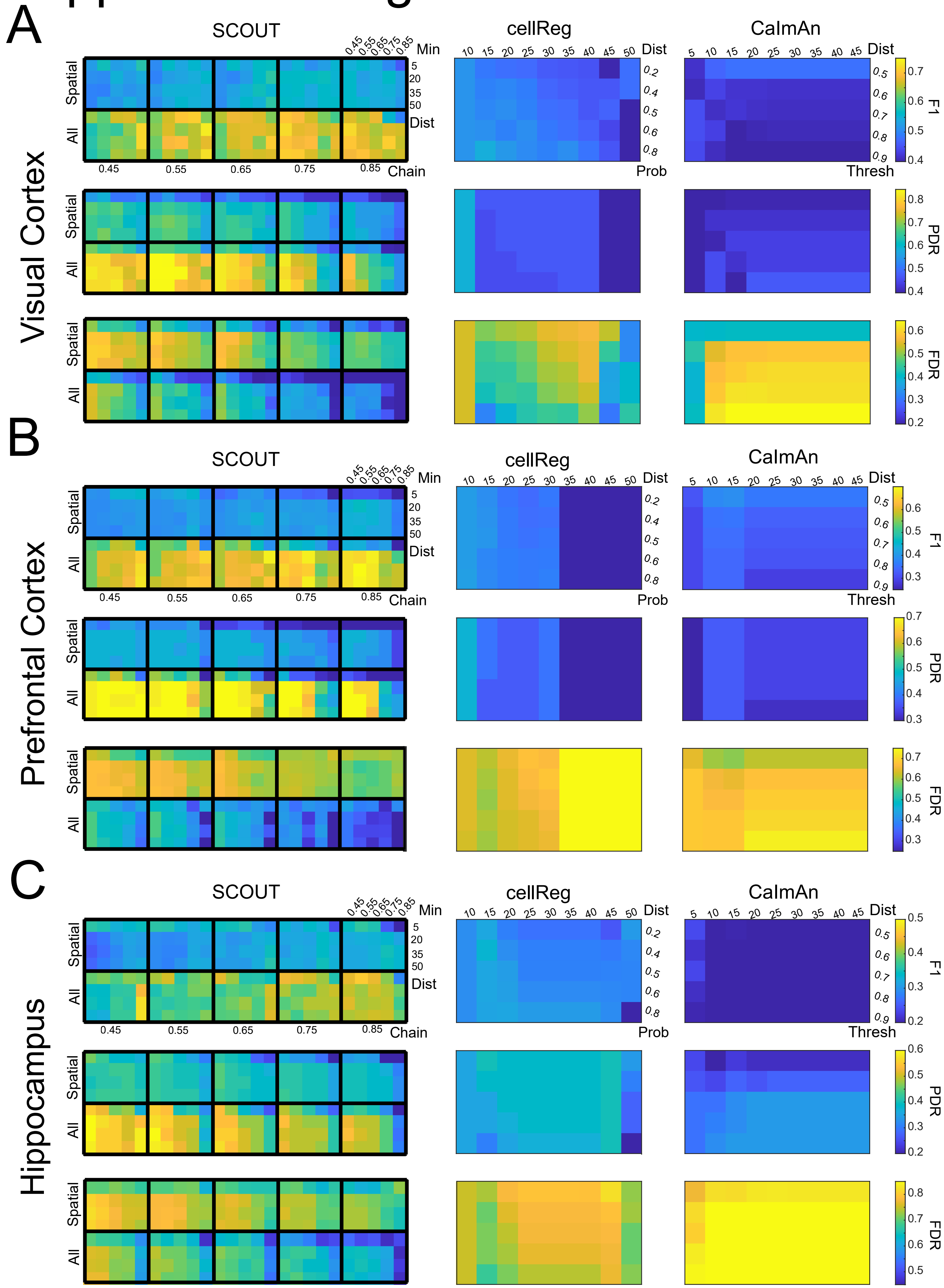

Supplemental Figure 5: **SCOUT cell tracking performance on 1-photon *in vivo* recordings, Related to Figure 4. A**: F1 scores (top), PDR (middle), and FDR (bottom) for SCOUT, cellReg, and CaImAn across a variety of parameters (labeled top), for the visual cortex recording. **B**: F1 scores (top), PDR (middle), and FDR (bottom) for SCOUT, cellReg, and CaImAn across a variety of parameters (labeled top) for the prefrontal cortex recording. Multiple cellReg parameters resulted in errors, the associated F1 and PDR were set to 0, and FDR to 1. **C**: F1 scores (top), PDR (middle), and FDR (bottom) for SCOUT, cellReg, and CaImAn across a variety of parameters (labeled top) for the hippocampus recording.

Supplemental Figure 6

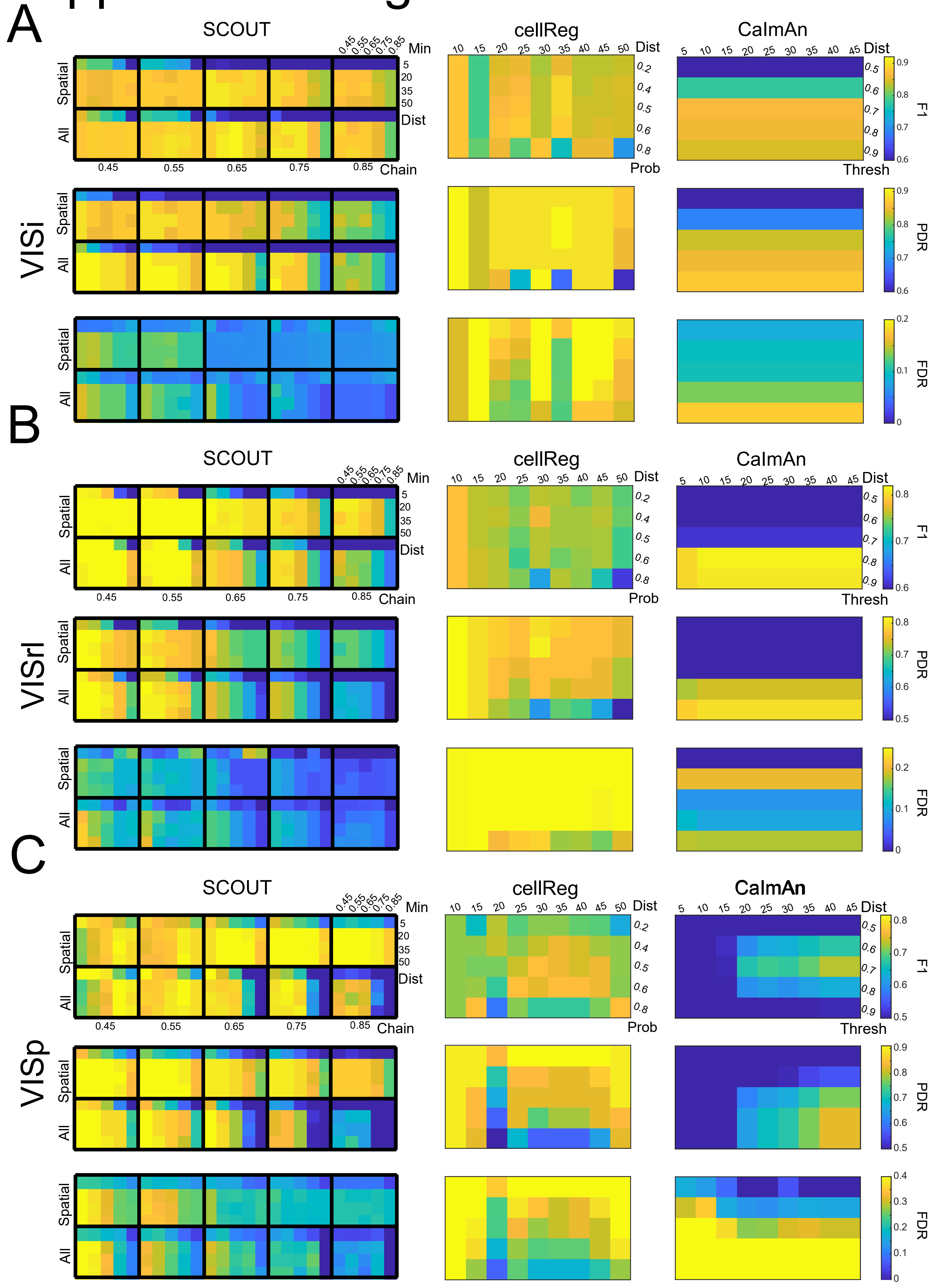

Supplemental Figure 6: **SCOUT cell tracking performance on the 2-photon *in vivo* recordings, Related to Figure 5**. **A**: F1 scores (top), PDR (middle), and FDR (bottom) for SCOUT, cellReg, and CaImAn across a variety of parameters (labeled top) for the VISi recording. **B**: F1 scores (top), PDR (middle), and FDR (bottom) for SCOUT, cellReg, and CaImAn across a variety of parameters (labeled top) for the VISrl recording. **C**: F1 scores (top), PDR (middle), and FDR (bottom) for SCOUT, cellReg, and CaImAn across a variety of parameters (labeled top) for the VISp recording.

Supplemental Table 1

		Gaussian	*Non-Rigid 1p*	*Non-Rigid 2p*	*Ind. Shift*
SCOUT	F1 Score	*0.986 +/- 0.005*	***0.970 +/- 0.003***	0.956 +/- 0.005	***0.707 +/- 0.013***
Spatial	F1 Score	*0.967 +/- 0.007*	0.929 +/- 0.005	0.949 +/- 0.005	0.526 +/- 0.010
cellReg	F1 Score	*0.964 +/- 0.007*	0.916 +/- 0.005	0.938 +/- 0.006	0.500 +/- 0.016
CalmAn	F1 Score	0.876 +/- .0131	0.924 +/- 0.004	0.953 +/- 0.006	0.547 +/- 0.015
SCOUT	Jaccard	*0.97 +/- 0.004*	***0.93 +/- 0.002***	0.93 +/-0.006	***0.78 +/- 0.011***
cellReg	Jaccard	*0.97 +/- 0.005*	0.90 +/- 0.003	0.92 +/- 0.006	0.68 +/- 0.008
CalmAn	Jaccard	0.88 +/- 0.006	0.90 +/- 0.003	0.93 +/- 0.007	0.68 +/- 0.007

Supplemental Table 1: F1 and Jaccard statistics for simulated data, Related to Figure 3. (upper) Average F1 score for each dataset and method. **Bold** entries indicate statistically significant improvement over cellReg. *Italicized* entries indicate statistically significant improvement over CaImAn. *Spatial* indicates SCOUT without temporal similarity metrics. (lower) Average Jaccard similarity accessing the ability of each method in identifying neurons for each dataset. **Bold** entries indicate statistically significant improvement over cellReg. *Italicized* entries indicate statistically significant improvement over CaImAn.
