# Supplementary material for: Tracking longitudinal population dynamics of single neuronal calcium signal using SCOUT
Source: Cell Rep Methods. 2022 Apr 29;2(5):100207. doi: 10.1016/j.crmeth.2022.100207 (PMC9142684; doi:10.1016/j.crmeth.2022.100207)
Supplement: Document S2. Article plus supplemental information [file mmc3.pdf]

Cell Reports
Methods

Article

# Tracking longitudinal population dynamics of single neuronal calcium signal using SCOUT

## Graphical abstract

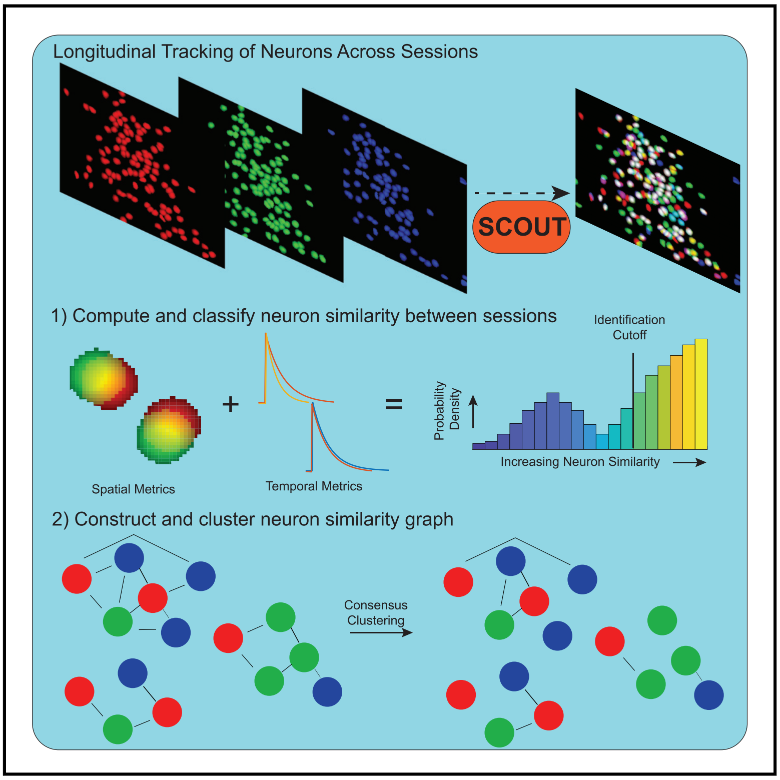


## Highlights

- SCOUT: a probabilistic model to track cells across multiple calcium imaging sessions
- Incorporates spatial and temporal metrics to compute cell-cell similarity probabilities
- Constrained consensus clustering for fast identification of neurons
- Demonstrates applicability to both 1- and 2-photon imaging recordings

## Authors

Kevin G. Johnston, Steven F. Grieco, Hai Zhang, Suoqin Jin, Xiangmin Xu, Qing Nie

## Correspondence

xiangmin.xu@uci.edu (X.X.), qnie@uci.edu (Q.N.)

## In brief

Johnston et al. develop SCOUT (Single-Cell spatiOtemporal longitUdinal Tracking), a method for tracking individual neurons across multiple $Ca^{2+}$ imaging sessions using both spatial and temporal metrics. SCOUT is designed for cell tracking in situations where strong spatial variations between sessions produce confounding factors that reduce the consistency of spatial features.

Johnston et al., 2022, Cell Reports Methods *2*, 100207
May 23, 2022 © 2022 The Authors.
https://doi.org/10.1016/j.crmeth.2022.100207

CellPress

Cell Reports Methods

CellPress
OPEN ACCESS

Article

# Tracking longitudinal population dynamics of single neuronal calcium signal using SCOUT

Kevin G. Johnston,[1] Steven F. Grieco,[2] Hai Zhang,[2] Suoqin Jin,[1] Xiangmin Xu,[2,4,5,6,7,*] and Qing Nie[1,3,4,7,8,*]
[1]Department of Mathematics and the NSF-Simons Center for Multiscale Cell Fate Research, University of California, Irvine, CA 92697, USA
[2]Department of Anatomy and Neurobiology, School of Medicine, University of California, Irvine, CA 92697, USA
[3]Department of Developmental and Cell Biology, University of California, Irvine, CA 92697, USA
[4]Department of Biomedical Engineering, University of California, Irvine, CA 92697, USA
[5]Department of Computer Science, University of California, Irvine, CA 92697, USA
[6]The Center for the Neurobiology of Learning and Memory, University of California, Irvine, CA 92697, USA
[7]The Center for Neural Circuit Mapping, University of California, Irvine, CA 92697, USA
[8]Lead contact
*Correspondence: xiangmin.xu@uci.edu (X.X.), qnie@uci.edu (Q.N.)
https://doi.org/10.1016/j.crmeth.2022.100207

**MOTIVATION** This work was motivated by the need for a cell-tracking technique for multi-session 1-photon imaging datasets. Our approach is robust to spatial footprint shifts and is able to accurately track cells in regions of overlapping footprints when multiple cells are possible targets. We discovered that incorporating cell-cell similarity metrics based on the temporal calcium activity of the detected neurons increases the number of correctly tracked cells and reduces the percentage of incorrectly tracked cells. Using this information, we improved the quality of the cell-cell similarity matrix and developed a method for tracking based on metric weight perturbation and consensus clustering.

## SUMMARY

*In vivo* calcium imaging enables simultaneous recording of large neuronal ensembles engaged in complex operations. Many experiments require monitoring and identification of cell populations across multiple sessions. Population cell tracking across multiple sessions is complicated by non-rigid transformations induced by cell movement and imaging field shifts. We introduce SCOUT (Single-Cell spatiOtemporal longitUdinal Tracking), a fast, robust cell-tracking method utilizing multiple cell-cell similarity metrics, probabilistic inference, and an adaptive clustering methodology, to perform cell identification across multiple sessions. By comparing SCOUT with earlier cell-tracking algorithms on simulated, 1-photon, and 2-photon recordings, we show that our approach significantly improves cell-tracking quality, particularly when recordings exhibit spatial footprint movement between sessions or sub-optimal neural extraction quality.

## INTRODUCTION

Extracting longitudinal activity from large-scale neuronal ensembles is a fundamental first step toward the analysis of neural circuit responses. $Ca^{2+}$ imaging of population neurons allows the recording of larger neural ensembles than can be recorded using electrophysiology. *In vivo* calcium imaging using microendoscopic lenses enables imaging of previously inaccessible ensembles of neuronal populations at the single-cell level in freely moving mice as they perform neural transformations that underlie behavioral responses over both short and long timescales (Flusberg et al., 2008; Ghosh et al., 2011; Ziv and Ghosh, 2015). Microendoscopic *in vivo* brain imaging via head-mounted fluorescent miniature microscopes ("miniscopes") are used widely to study neural circuits in various brain regions (Cai et al., 2016; Ziv et al., 2013; Jimenez et al., 2016; Rubin et al., 2015; Sun et al., 2019; Kitamura et al., 2015; Sun et al., 2015; Barbera et al., 2016; Klaus et al., 2017; Yu et al., 2017).

Experiments that require the accurate identification of neurons across multiple recording sessions have proved difficult, as cell movement, shifts in field of view, and inaccuracies in the extraction of neural activity from session recordings complicate this task. Most previous attempts to track the activity of neurons over long-term experiments have taken one of three forms (Figures 1A and 1B): (1) initial concatenation of registered recordings followed by extraction of fluorescence traces and spatial footprints from the concatenated recording (Sun et al., 2019); (2) concatenation followed by splitting the spatial dimension into overlapping patches, whereby extraction is performed on each patch separately and neurons are merged across the


This is an open access article under the CC BY license (http://creativecommons.org/licenses/by/4.0/).

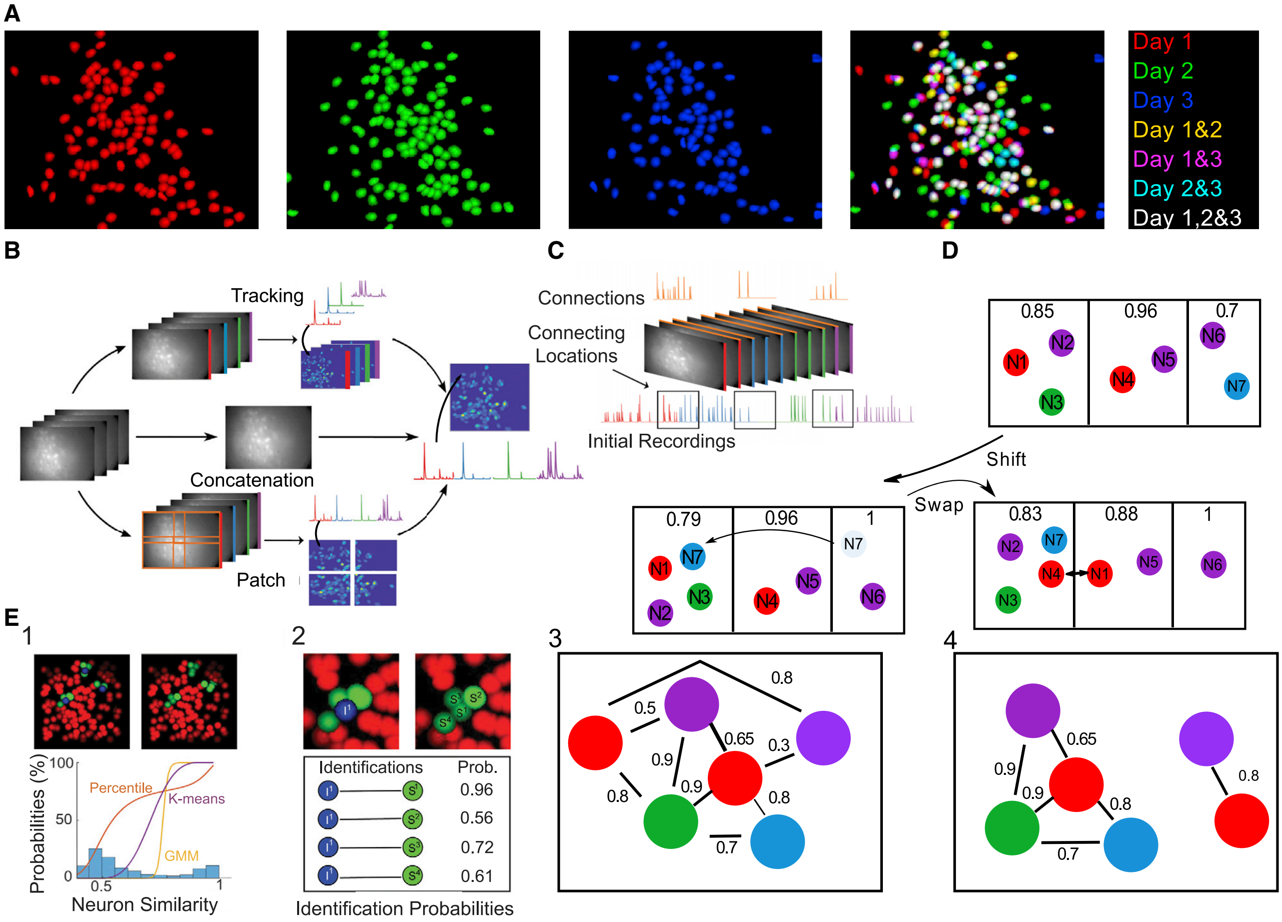


**Figure 1. SCOUT: A method for single-cell tracking incorporates spatial and temporal metrics into a probabilistic consensus clustering framework**

(A) Neuron spatial footprints from three sessions obtained from a 1-photon recording of the prefrontal cortex with neurons colored by session (first three rectangles). Overlaid results appear in the fourth rectangle. Cell tracking seeks to identify the same cell across multiple days.

(B) Long-term study of neural activity requires computation of fluorescence traces for identified neurons across sessions (right) from individual sessions (max projections, left). Approaches include concatenation (middle track), patch methods (bottom track), and tracking methods (top track). Concatenation involves global registration of sessions and concatenation (middle step) followed by fluorescence extraction. Patch methods divide each session into overlapping patches in the spatial domain (orange rectangles, first step), which are concatenated, and fluorescence activity extracted for each neuron (black arrow, second step) followed by merging patches. Tracking methods extract traces for all neurons in each session (first step), followed by identification of neurons across sessions (second step).

(C) Temporal correlation involves a link session (orange) between consecutive pairs of recordings. High-quality neurons result in a corresponding neuron in the link session with matching neural signals used to identify cells across sessions.

(D) SCOUT clustering algorithm groups cells from different sessions into clusters. Boxes indicate separate clusters, with the color of each circle indicating the session. The associated numbers indicate the within-cluster similarity for the given cluster.

(E) Demonstration of the SCOUT algorithm. (1) Several cells (blue) and their neighbors (green) within a session (top left) and between sessions (top right). Histogram of cell-cell similarity between sessions for a metric (bottom) with overlaid identification likelihood using several models. (2) A single cell (blue) and its neighbors (top) within and between sessions with sample aggregate (across metrics) identification probabilities (bottom). (3) A sample graph in which nodes indicate neurons, and edges between nodes denote identification probability exceeding a minimum threshold (*min_prob*). Colors correspond to sessions. (4) A possible graph clustering.

patches, giving extracted footprints and neural signals throughout the full recording (Zhou et al., 2018); (3) extraction of neural signals from each session independently, followed by tracking cells across recordings via spatial similarities in the extracted neuron footprints (Sheintuch et al., 2017; Cai et al., 2016).

Concatenation and patch methods can be resource intensive in terms of both computational power and time while requiring neuron spatial footprints to remain in constant position over time. In terms of scalability, cell-tracking methods may provide the best option for long-term neural ensemble analysis, but several factors complicate the cell-tracking task.

First, imperfect motion correction or low signal-to-noise ratio (SNR) can reduce the quality of neuron extractions, leading to false discoveries (neurons identified by extraction algorithms

corresponding to noise or motion artifacts). Second, global registration can lead to varying centroid distances and overlap between identified neurons across the field of view (FOV). Third, variability in neuron position and/or FOV changes can reduce tracking accuracy across sessions. Finally, some analyses require analysis of neural signals across all sessions, in which case lowering the detection threshold for neuron extraction may allow for the identification of lower signal neurons at the cost of an increased false discovery rate (FDR) (Video S1). These factors are compounded when experiments take place over extended time periods (>30 sessions). An ideal cell-tracking algorithm should therefore be robust to changes in neuron position, false discoveries, and missing neurons. To address these issues, spatial metrics alone are insufficient.

We present SCOUT (Single-Cell spatiOtemporal longitUdinal Tracking), a method for tracking individual neurons across multiple sessions using both spatial and temporal metrics. SCOUT uses the temporal similarity metrics of SNR and fluorescence decay rate, as well as a new correlation metric that uses connecting recording segments to verify the neuron identification (STAR Methods and Figure 1C) in addition to standard spatial metrics such as centroid distance, footprint overlap, and Jensen-Shannon (JS) divergence (Kullback, 1997), to improve neuron identification between sessions. SCOUT also provides the option of allowing the user to define additional metrics according to their use case. SCOUT then uses a combination of probabilistic models, a novel clustering algorithm, and consensus clustering to perform cell tracking over multiple sessions (Figures 1D and 1E). This combination of features makes SCOUT unique among cell-tracking methods.

## RESULTS

### SCOUT: A single-cell multi-session tracking algorithm incorporates both spatial and temporal metrics

SCOUT cell tracking consists of four steps: (1) cell-cell identification probability computation for session pairs; (2) creation of cell-cell similarity matrices; (3) clustering of the cell-identification matrices and consensus clustering of the resulting cell identifications; (4) creation of a cell register defining indices of identified cells between sessions and associated neural signals (Figure 1E).

(1) For each metric, SCOUT selects all neuron pairs having non-zero spatial overlap and footprint centroids within a user-specified distance (*max_dist*). Next SCOUT constructs a probabilistic model (STAR Methods) dividing the resulting similarity values into those corresponding to identified cells between sessions, and those corresponding to overlapping but non-identified cells, assigning identification probabilities to each pair of cells.

(2) SCOUT perturbs a *weights* vector governing the importance of each metric and creates an aggregated cell-cell probability by weighting the cell identification probabilities in step (1). Applying this to each pair of sessions creates a unique similarity matrix corresponding to each weight, containing the cell-cell identification probabilities. These matrices have size (n_cells × n_cells), where n_cells is the combined total number of neurons in all sessions. Cells with identification probability below a threshold (*min_prob*) have similarity set to zero.

(3) SCOUT applies a tailor-made clustering algorithm to each similarity matrix. Clusters are constrained by the number of sessions and the requirement that neurons from the same session belong to different clusters. At each iteration of the clustering algorithm, SCOUT computes the total increase in average similarity between cluster members (over all clusters) gained by assigning each neuron to a new cluster (a switch) or by swapping the cluster assignments of each pair of neurons (a swap) (Figure 1D). The operation that maximizes the similarity increase is accepted, and the algorithm continues until a maximum number of iterations is reached or no further gains can be made by these operations. Finally, if the minimal average cluster similarity falls beneath a *chain_prob* parameter, a new cluster is created and populated with the least similar neuron in the lowest scoring cluster (ranked by average within-cluster similarity), and the process repeats until convergence. Dissimilarity-based cluster initialization and induced bias toward larger cluster sizes increases accuracy and ensures convergence (STAR Methods). SCOUT constructs a consensus probability matrix based on the clustering results from the previous section. The $(i,j)$ entry gives the probability that the $i^{th}$ and $j^{th}$ neurons belong to the same cluster, based on the outputs in step (2) computed for each similarity matrix. Applying the previously described clustering algorithm to this consensus matrix creates finalized cluster identifications.

(4) Cell clusters are placed in a cell register, a matrix in which each row corresponds to a tracked neuron and each column to a session. Calcium traces corresponding to each tracked neuron are concatenated to obtain a neural signal that traverses each session in the recording.

In this paper we explicitly demonstrate that temporal metrics are consistent within and across sessions, justifying their inclusion in the algorithm. We then demonstrate the effectiveness of SCOUT on a set of simulated datasets, consisting of the Gaussian dataset (control dataset, low background noise levels; Figure S1A and STAR Methods), the Non-Rigid 1-photon (1p) dataset (variable sized spatial footprints warped in place by generated non-rigid transformation, high background noise levels; Figure S1B), the Non-Rigid 2-photon (2p) dataset (modified version of the Non-Rigid 1p dataset in which footprint centers are removed to simulate characteristic ring shapes, salt and pepper noise; Figure S1C), and the Individual Shift dataset (spatial footprints individually translated a random distance, no background noise; Figure S1D). We also test SCOUT on 1-photon experimental data from the visual cortex (VC) (Grieco et al., 2020), the prefrontal cortex (PFC) (Grieco et al., 2021), and the hippocampus (Hipp) (Sun et al., 2019) (Figure S1E), which exhibit strong variation in neural signals due to experimental conditions (STAR Methods), as well as three 2-photon recordings taken from the visual cortex (Allen, 2016) labeled VISl, VISrl, and VISp (Figure S1F), taken as head-fixed mice were

shown various stimuli (STAR Methods) causing neural signal variability. Finally we test place-field stability of neurons identified by SCOUT on three 1-photon recordings taken from the hippocampus. Each dataset presents unique difficulties for cell-tracking algorithms to address.

## Usage of temporal metrics improve discrimination between identified and non-identified neurons across sessions

We first show that the temporal metrics SNR, decay rate, and temporal correlation are consistent across and within sessions and provide additional discriminatory features useful for identifying neurons across sessions (Figures 2A–2C). To demonstrate within-session consistency, we split the first session of *in vivo* recording sessions in half longitudinally and compute SNR and decay rate metrics on the resulting sessions. For each neuron in the first half, we compute the absolute difference of the decay rate and SNR for the same neuron in the second half, and the nearest non-identified neuron in the second half as comparison (Figures 2D and 2E).

Ratios of the median difference between the same and nearest-neighbor neurons are significantly lower than 1.0 ($p = 2.9 \times 10^{-33}$, $1.5 \times 10^{-4}$, $1.2 \times 10^{-10}$ for VC, PFC, Hipp, respectively, Wilcoxon rank-sum test), as are median SNR difference ratios ($p = 2.3 \times 10^{-24}$, $6.9 \times 10^{-4}$, $4.8 \times 10^{-3}$). Both SNR and fluorescence decay exhibit slightly higher ratios on 2-photon data, although this effect is primarily due to a single recording (decay: $p = 0.02$, 0.04, 0.04; SNR: $p = 1.4 \times 10^{-7}$, $4.7 \times 10^{-2}$, $7.4 \times 10^{-10}$ for VISl, VISrl, VISp, respectively). Ratios ranged from 0.5 to 0.65 (i.e., 50%–65% of the difference of non-identified nearest neighbors), indicating much lower differences between SNR and decay rate of the same neuron compared with its neighbors.

Next, we compute similarity of temporal metrics (including correlation metric) between neurons in the first session (unsplit) and the second session of *in vivo* recordings, to demonstrate that temporal metrics discriminate between identified and non-identified neurons between sessions. This context adds complications, as some neurons may not correspond to identified pairs in the other session, and the ground truth is unknown. We assume that the most similar (based on the current temporal metric) neurons with overlap exceeding 0.9 are identified, which are compared with the nearest neighbor below this threshold. Neurons in the first session with no other spatial footprints within four pixels in the second session are excluded from the analysis.

On 1-photon data, median absolute difference in decay rates is significantly lower ($p = 4.1 \times 10^{-9}$, $8.8 \times 10^{-5}$, $2.4 \times 10^{-5}$ for VC, PFC, Hipp, respectively, Wilcoxon rank-sum test), as are differences in SNR ($p = 6.3 \times 10^{-8}$, $2.5 \times 10^{-4}$, $3.1 \times 10^{-6}$), with similar results on 2-photon recordings ($p = 0.006$, 0.006, $6.3 \times 10^{-9}$ for VISl, VISrl, VISp, respectively). One result did not show significant SNR differentiation ($p = 0.98$, 0.03, 0.01). Average correlation metric values on both 1-photon data ($p = 2.2 \times 10^{-22}$, $2.2 \times 10^{-8}$, $2.8 \times 10^{-9}$) and 2-photon data ($p = 7.2 \times 10^{-5}$, $1.1 \times 10^{-3}$, $6.8 \times 10^{-12}$) show higher median temporal correlation for identified versus non-identified neurons.

Ratios of median temporal metric difference are higher between sessions (i.e., more difference between sessions than within sessions), with differences between 60% and 75% those of nearest neighbors for SNR and decay, and with correlation ~1.5 times higher between identified neurons and nearest neighbors. This implies that the median pair of identified neurons has significantly more similar temporal profiles than nearest neighbors, which motivates the inclusion of temporal metrics in SCOUT.

## Testing SCOUT on simulated multi-session recordings

On simulated recordings we first consider only neurons identified through all sessions, as these are the most easily interpreted in downstream analysis. Cell-tracking quality is defined using the F1 metric $\frac{2 \times PDR \times (1 - FDR)}{(1 - FDR + PDR)}$, which takes values between 0 and 1 (1 being the highest quality). Here, percent discovery rate (PDR) is defined as the percentage of available neurons tracked by a method, and FDR is defined as the percentage of tracked neurons containing at least one false identification. Next, we consider tracking quality for all sets of identified neurons using the Jaccard similarity metric (computed as $|A \cap B| / |A \cup B|$, where $A$ and $B$ represent sets of identified neurons). This analysis includes all clusters of identified neurons. For testing purposes, we use neuron footprint centroid distance, spatial overlap, and JS divergence (spatial metrics), and SNR, fluorescence decay rate, and correlation (temporal metrics).

We test SCOUT (with and without temporal metrics), cellReg, and CaImAn multiple times on each dataset with varying parameters (*max_dist*, *min_prob*, *chain_prob* for SCOUT, see STAR Methods, and Figures S2 and S3 for cellReg and CaImAn parameters). Here we present results from parameters maximizing the F1 score across each tested method. Statistical results are computed using ANOVA and post hoc Bonferroni correction (Bonferroni 1936) for multiple comparisons. Quantitative results are presented as mean ± SE where each data point corresponds to a single recording in the dataset.

We identify significant F1 score differences on the Gaussian, Non-Rigid 1p, and Individual Shift datasets (Gaussian: $p = 3.06 \times 10^{-12}$, $F = 40.77$; Non-Rigid 1p: $p = 1.87 \times 10^{-16}$, $F = 32.92$; Individual Shift: $p = 3.35 \times 10^{-19}$, $F = 45.14$; ANOVA). Pairwise comparisons show a statistically higher average F1 score between SCOUT and CaImAn on the Gaussian dataset (Figures 3A and S1A–S1C), a higher average F1 score between SCOUT and both cellReg and CaImAn on the Non-Rigid 1p dataset (Figures 3B and S1D–S1F), no significant differences on the Non-Rigid 2p dataset (Figures 3C and S2A–S2C), and a higher F1 score for SCOUT than both cellReg and CaImAn on the Individual Shift dataset (Figures 3D and S2D–S2F) (CaImAn comparisons $p < 1.16 \times 10^{-11}$, cellReg comparisons $p < 2.6 \times 10^{-17}$). Additionally, SCOUT with temporal metrics outperforms SCOUT without temporal metrics on both the Non-Rigid 1p dataset and the Individual Shift dataset ($p < 3.5 \times 10^{-9}$).

In summary, SCOUT exhibits high-quality cell-tracking performance when compared with methods such as cellReg and CaImAn, particularly on the Non-Rigid 1p and Individual Shift datasets (Table S1; Figures S2 and S3). Comparisons of SCOUT with and without temporal metrics show that inclusion of temporal metrics in the analysis results in higher average F1 scores.

We next compute Jaccard similarity on the same data. This method identifies significant differences in mean tracking quality

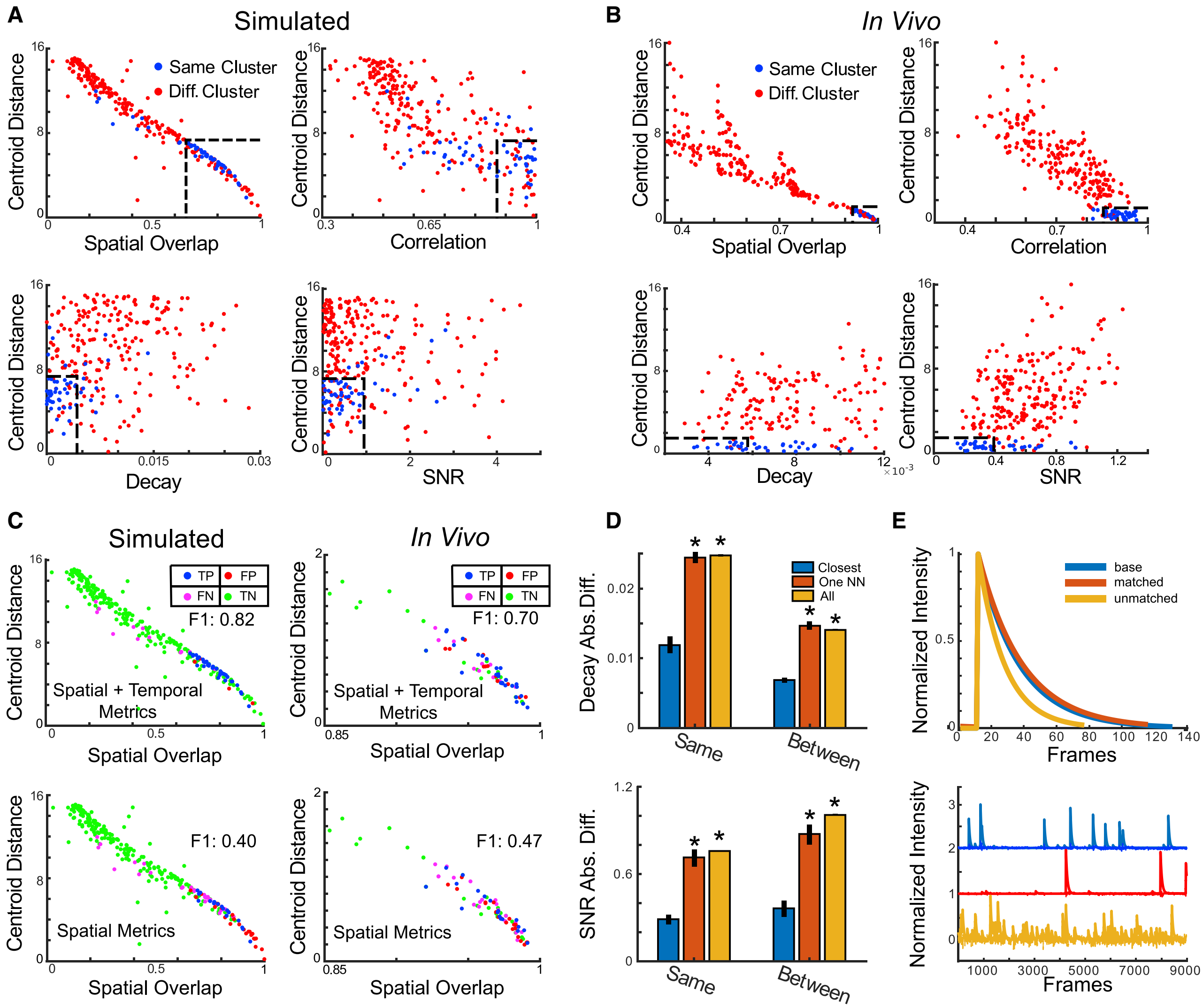


**Figure 2. Inclusion of temporal metrics is a key step when there is significant shift between neurons across sessions**

(A) Scatterplots compare spatial (overlap) and temporal (SNR, decay, correlation) metrics (x axis) with the centroid distance metric (y axis) for all neuron pairs (identified, blue; non-identified, red; labeled by ground truth) from two sessions of a recording taken from the Individual Shift dataset. Approximate decision boundaries for each metric are indicated by the black dashed line.

(B) Scatterplots compare inter-cluster metric similarity on a 1-photon *in vivo* recording consisting of seven sessions. Correct identifications based on human annotated ground truth cell register. Incorrect identifications are simulated by randomly exchanging a neuron in a ground truth cluster with a near neighbor (as measured by centroid distance). Plotted points indicate average similarity (by metric) for both correct (blue) and incorrect (red, at least one error) clustering results. Approximate decision boundaries for each metric are indicated by the black dashed line.

(C) Similarity metrics (as shown in A and B) are aggregated with resulting cell similarities used for clustering. False positives (FP), true positives (TP), false negatives (FN), and true negatives (TN) are labeled in red, blue, purple, and green, respectively. Results using all metrics (top) and exclusively spatial metrics (bottom), presented for the simulated (left) and *in vivo* (right) recordings.

(D) Bar charts compare SNR and decay metrics between identified neurons, nearest neighbors, and average similarity across all neurons, within and between two sessions of an *in vivo* 1p recording. (Top) SNR absolute differences (y axis) after splitting the first recording into two sessions (same), and between sessions (between) for identified neurons (Closest), nearest neighbors (One NN), and all neuron pairs (All). (Bottom) Absolute signal decay rate differences within and between sessions. The reduction in value between sessions is due to the use of post-extraction computation decay for the within-session data. Error bars indicate SE across associated neuron pairs. Asterisks indicate significant differences between identified and nearest neighbors/All pairs using Wilcoxon rank sum test ($p < 5 \times 10^{-3}$).

(E) (Top) Neural traces associated with a single spike from three neurons taken from two sessions of an *in vivo* 1-photon recording: (blue) a baseline neuron from the first session, (red) an identified neuron from the second session, (yellow) a non-identified neuron from the second session. (Bottom) Neural traces from the baseline, identified, and non-identified neurons along with the noise level after normalization to unit peak intensity.

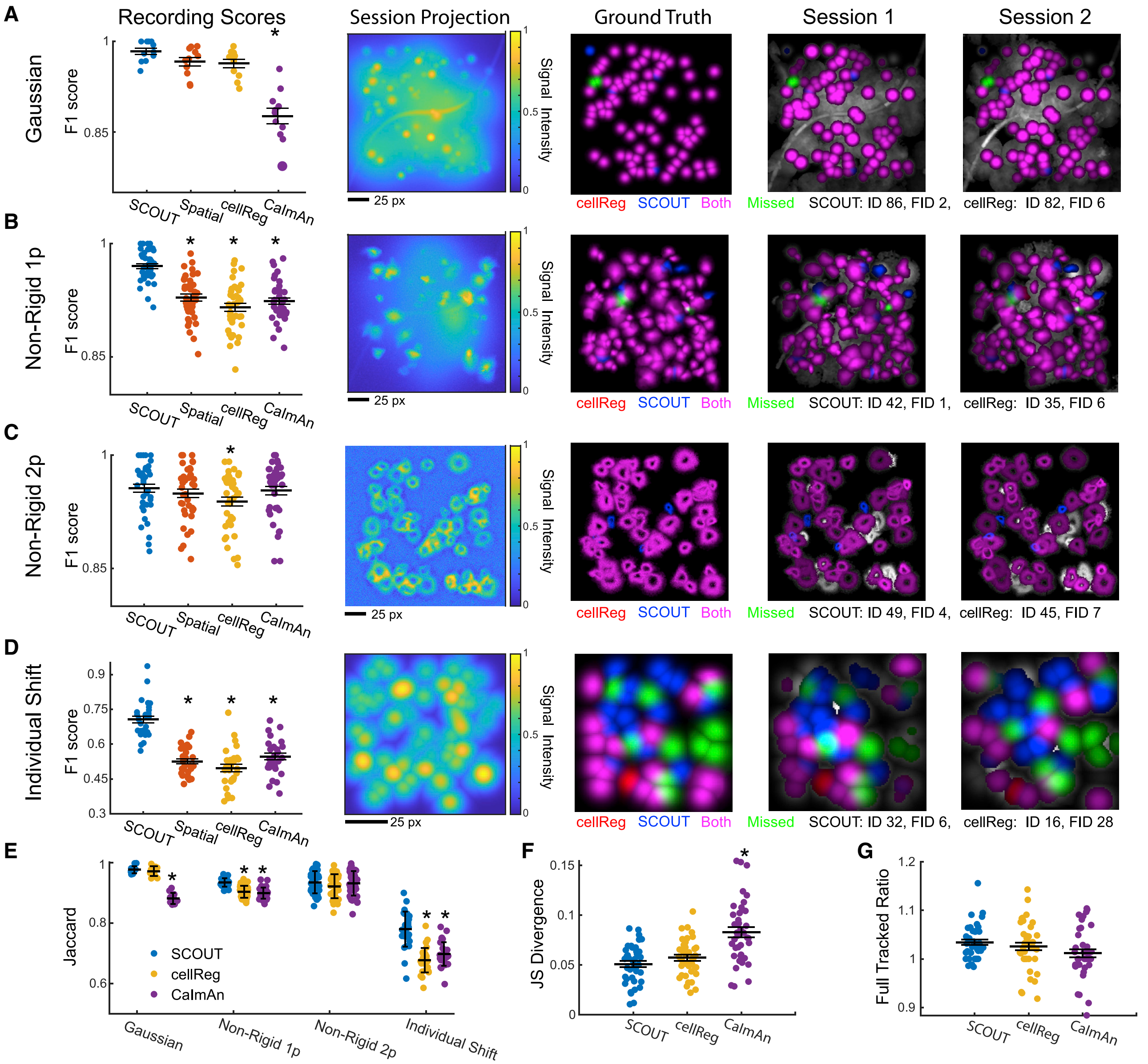


**Figure 3. Inclusion of temporal similarity metrics improves cell tracking across sessions on simulated datasets**

(Recording Scores) Maximal F1 scores (y axis) using all tested cell tracking algorithms. Statistically significant differences compared with SCOUT (ANOVA, Bonferroni correction) are marked with an asterisk. Methods (x axis) are SCOUT, spatial (SCOUT using only spatial metrics), cellReg, and CalmAn. (Session Projection) Max projection of sample individual session from each dataset, across methods. (Ground Truth) Ground truth neurons available for tracking through all sessions. Colors indicate neurons tracked by each method. The number of correctly tracked (ID) and incorrectly tracked with at least one error (FID) cell register entries are labeled below for each method. (Sessions 1 and 2) Tracked and missed neurons superimposed on the max projection of extracted neurons from sessions 1 and 2 of a sample recording. For all panels, bars indicate mean ± SE and asterisks indicate statistical significance of pairwise comparisons with SCOUT (ANOVA, Bonferroni correction for multiple comparisons).

(A–D) F1 scores, session projections, ground truth, and identified neurons by method for the Gaussian (A), Non-Rigid 1p (B), Non-Rigid 2p (C), and Individual Shift (D) datasets.

(E) Maximal Jaccard similarity scores (y axis) from each recording and session in the simulated datasets (x axis). Bars indicate mean ± SE. Asterisks indicate statistically significant differences.

(F) JS divergence (y axis) of identified cluster sizes for each method (x axis) with the ground truth for each recording in the Non-Rigid 1p dataset using parameters producing highest F1 scores.

(G) y axis: the ratio of neurons tracked through all sessions by each method to the ground truth number of neurons available for tracking using parameters producing highest F1 scores.

See also Figures S2 and S3; Table S1.

on all datasets except for the Non-Rigid 2p dataset (Gaussian: $p = 2.1 \times 10^{-15}$, $F = 127.8$; Non-Rigid 1p: $p = 1.64 \times 10^{-15}$, $F = 46.6$; Individual Shift: $p = 7.8 \times 10^{-13}$, $F = 39.6$; ANOVA, Figure 3E). Post hoc Bonferroni tests show that SCOUT exhibits higher Jaccard similarity than CaImAn on the Gaussian dataset ($p = 2.2 \times 10^{-14}$), higher Jaccard similarity than both CaImAn and cellReg on the Non-Rigid 1p dataset ($p < 1.4 \times 10^{-11}$), and higher Jaccard similarity than both cellReg and CaImAn on the Individual Shift dataset ($p < 4.8 \times 10^{-9}$). These results correspond with F1 score results and show that SCOUT exhibits higher cell-tracking quality across all proposed clusters, not just neurons tracked across all sessions (Table S1).

To identify possible biases toward large cluster sizes with SCOUT, we compute the JS divergence between projected cluster distributions and ground truth cluster distributions for each method on the Non-Rigid 1p dataset (the dataset exhibiting the largest difference with more than two sessions per recording), using the parameters giving the best average F1 score for each method (Figure 3F). ANOVA shows significant differences between average JS divergence across methods ($p = 4.5 \times 10^{-9}$, $F = 22.9$), with post hoc comparisons exhibiting lower average JS divergence for SCOUT compared with CaImAn ($p < 2.5 \times 10^{-9}$; JS: SCOUT $0.051 \pm 0.003$, cellReg $0.057 \pm 0.003$, CaImAn $0.083 \pm 0.005$). Computing the ratio of neurons tracked through all sessions for each method to the ground truth demonstrates comparable results for all methods when considering the statistic $abs(1 - ratio)$ (Figure 3G).

We next compute Jaccard similarity, JS distribution similarity, and percentage of tracked neurons after removing 30% of neurons from each session (and the associated ground truth cell register) to determine whether significantly reducing the available neurons per session affected cell-tracking quality. This analysis shows that SCOUT exhibits significantly higher similarity with the ground truth distribution compared with cellReg and CaImAn (ANOVA: $F = 22.8$, $p = 4.9 \times 10^{-9}$, pairwise comparisons $p < 0.03$) while exhibiting lower overdetection rates of neurons through all sessions when compared with cellReg (ANOVA: $F = 5.9$, $p = 3.6 \times 10^{-3}$, pairwise comparisons $p = 0.020$, statistic $abs(1 - ratio)$) (Figures S4A–S4C). This implies that inferred tracking registers produced by SCOUT exhibit close similarity to ground truth distribution on similar scales with other methods, while also being robust to neuron deletion.

Next, we repeatedly perform cell tracking with SCOUT using the spatial metrics and a single temporal metric on the Non-Rigid 1p and Individual Shift datasets, as these datasets exhibit significant variation upon inclusion of temporal metrics. On the Non-Rigid 1p dataset, inclusion of each individual additional temporal metric results in higher average F1 scores, with similar results on the Individual Shift dataset, except for the SNR metric, which is expected because no background noise is simulated in this dataset (Figures S2D–S2F and S3D–S3F). Combining temporal metrics increases the average F1 score in most instances.

Finally, we run speed tests (24 core, 128 GB pc, 2.2 GHz processor) by duplicating simulated data sessions to produce recordings with up to 30 sessions. We compare SCOUT, cellReg, and CaImAn on 50 simulated recordings. Both cellReg and SCOUT exhibit quadratic time increase in the number of sessions (Figure S4D), compared with linear time increase for CaImAn. However, the quadratic coefficient for SCOUT is lower than for cellReg, and SCOUT significantly outperforms cellReg in terms of runtime while maintaining comparable results with CaImAn for recordings of length up to 20 sessions.

Using both F1 and Jaccard metrics, SCOUT scores higher than alternative methods on the Non-Rigid 1p and Individual Shift datasets. JS divergence shows that the distribution of identified neuron cluster sizes is significantly closer to the ground truth using inferred cell registers from SCOUT, while the percentage of neurons tracked through all sessions is largest with SCOUT. Inclusion of temporal metrics increases average F1 score and shows that SCOUT typically runs faster than cellReg, with speed comparable with CaImAn for recordings of length up to 20 sessions.

## SCOUT successfully tracks cells on *in vivo* multi-session recordings

We evaluate SCOUT on *in vivo* 1-photon recordings (Figure S1E) taken from the visual cortex, prefrontal cortex, and hippocampus of mice consisting of 4–7 sessions (4,000–9,000 frames each) from each region, compared with annotated cell registers (see STAR Methods for annotation criteria).

On the visual cortex dataset, optimal parameters (among those tested) give F1 scores of 0.736 and 0.590, tested on SCOUT with and without temporal metrics, respectively. This resolves to PDR of 78.0% and 61.0% and FDR of 30.3% and 42.9% (Figures 4A and S5A). On the prefrontal cortex dataset, optimal parameters give F1 scores of 0.701 and 0.468, with PDR 71.1% and 47.4% and FDR 30.8% and 53.9% (Figures 4B and S5B). On the hippocampus dataset, optimal parameters give F1 scores of 0.481 and 0.367 with PDR of 58.5% and 35.4% and FDR of 59.2% and 62.0%, respectively (Figures 4C and S5C). Average F1 scores and PDR with SCOUT ($0.639 \pm 0.080$ and 69.2%, respectively) exceed those of cellReg ($0.431 \pm 0.107$ and 48.9%) and CaImAn ($0.368 \pm 0.123$ and 34,0%), and individually exceed both methods on all 1-photon datasets.

The three 2-photon recordings (Figure S1F) consist of three sessions, one taken from the VISl (Figures 5A and S6A), the VISrl (Figures 5B and S6B), and the VISp (Figures 5C and S6C). Optimal parameters give average F1 scores and PDR for SCOUT ($0.875 \pm 0.025$ and 91.1% with temporal metrics, $0.862 \pm 0.024$ and 89.5% without temporal metrics), cellReg ($0.816 \pm 0.038$ and 85.2%), and CaImAn ($0.803 \pm 0.04$ and 78.0%).

Together, SCOUT exhibits F1 scores $\sim$50% higher than cellReg and CaImAn on 1-photon data, while median place-field consistency for neurons identified exclusively by SCOUT is comparable with consistency of neurons identified by both methods on the three additional hippocampus datasets, and significantly lower than the consistency between random pairs of neurons. While the difference is smaller, SCOUT also produces top-ranked F1 scores on the 2-photon dataset.

## Testing performance via place cell stability analysis

We test SCOUT and cellReg on three additional 1-photon recordings of the hippocampus to verify cell-tracking results via

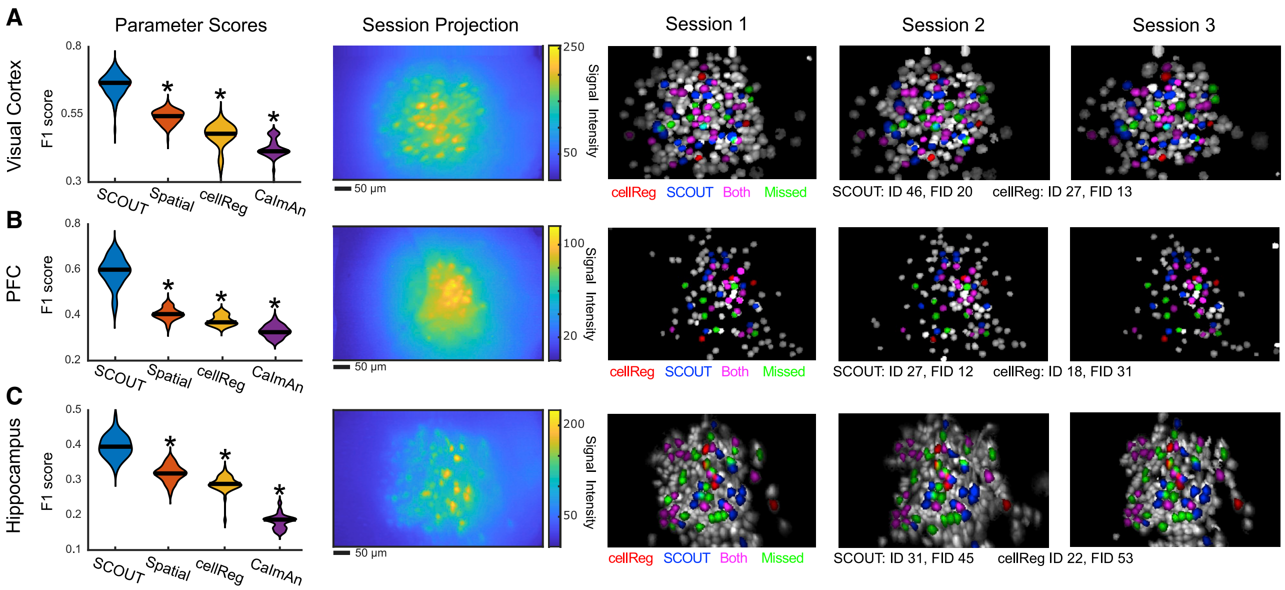


**Figure 4. Inclusion of temporal metrics boosts cell-tracking performance on 1-photon *in vivo* data**

(A–C) (Parameter Scores) F1 scores (y axis) computed based on human annotation for *in vivo* 1-photon datasets obtained from the visual cortex (A, seven sessions), prefrontal cortex (B, seven sessions), and hippocampus (C, four sessions). Violin plots with median values constructed using F1 scores across parameters after outlier removal and computed using kernel density estimation. Asterisks indicate statistically significant differences compared with SCOUT (ANOVA, Bonferroni). (Session Projection) Maximum projection of the first session of each recording from all datasets. (Sessions 1–3) Identified neurons from cellReg and SCOUT overlaid on max projection of the human annotated neurons tracked through all sessions.

See also Figure S5.

place-field stability. These recordings consist of four sessions with 10,795 frames each (at 30 Hz), taken as mice run on a 1-m linear track. Sessions are extracted via CNMF-E, and both SCOUT and cellReg are used to compute cell registers for each recording set, using the best average parameters on the 1-photon data from the previous analysis (see STAR Methods for exact values). For each neuron in each session, information scores and place fields are computed, and information percentiles are computed via random shuffling of the position vector.

To reduce noise, we remove all register entries consisting of neurons identified in only two sessions and analyze results for all identified cell pairs (e.g., a cell tracked through four sessions produces six identified cell pairs). We place identified cell pairs in three categories: cell pairs identified by both methods, cell pairs identified by SCOUT, and cell pairs identified by cellReg. This results in an average of 558 ± 208 pairs identified by both methods, 327 ± 182 pairs identified exclusively by cellReg, and 122 ± 48 pairs identified exclusively by SCOUT. The high variance is due primarily to fewer extracted neurons in the third recording.

For each cell pair, we compute statistics for average JS divergence between place fields, percentage of identified cells with JS divergence below assignment threshold (i.e., consistent place fields), and the percentage of identified neurons in which either place cells are matched with place cells, or non-place cells are matched with non-place cells. We analyze results at a variety of information percentiles (range [0.95, 0.99]) and information score thresholds (range [0.5, 1.5]). For each threshold, we restrict analysis to only neuron pairs in which at least one member exhibits information percentile or information score exceeding the specified thresholds. Prior to analysis, we verify for each recording that the average JS divergence rate for identified cells by both SCOUT and cellReg is significantly lower than the average between random cell pairs ($p < 2.6 \times 10^{-9}$, two-sample t test), implying that a significant number of identified neuron pairs exhibit place-field stability across sessions (Figure 6A).

Computing statistics for average JS divergence between place fields, percentiles exceeding 0.95 and information score thresholds in range $[0.8, 1.5]$ result in statistically lower JS divergence between place fields exclusively identified by SCOUT compared with those identified exclusively by cellReg (min p = 0.0079, max p = 0.0488, linear mixed effects model with fixed cell-tracking method grouped by recording, Figure 6B). This range of values matches that of a previous study (Grijseels et al., 2021) that suggests a percentile threshold of 0.95–0.99 of place cells, while experimentation showed that neurons with information score threshold exceeding 1.0 showed strong spatially localized firing activity.

Setting a threshold of 0.95 for percentile and 1.3 for info score threshold, we compute the percentage of identified neurons with JS divergence below an acceptance threshold (range [0.025, 0.1]) for neuron pairs containing place cells, a range that typically implies strong place-field overlap. For percentile and information score thresholds, SCOUT exhibits a larger fraction of identified neurons below the acceptance threshold across the entire range when compared with cellReg (average difference, percentile: 0.086 ± 0.065; threshold: 0.098 ± 0.06; mean ± SE, Figure 6C). Similarly, the fraction of SCOUT-identified pairs is more likely than those identified by cellReg to identify place cells with place

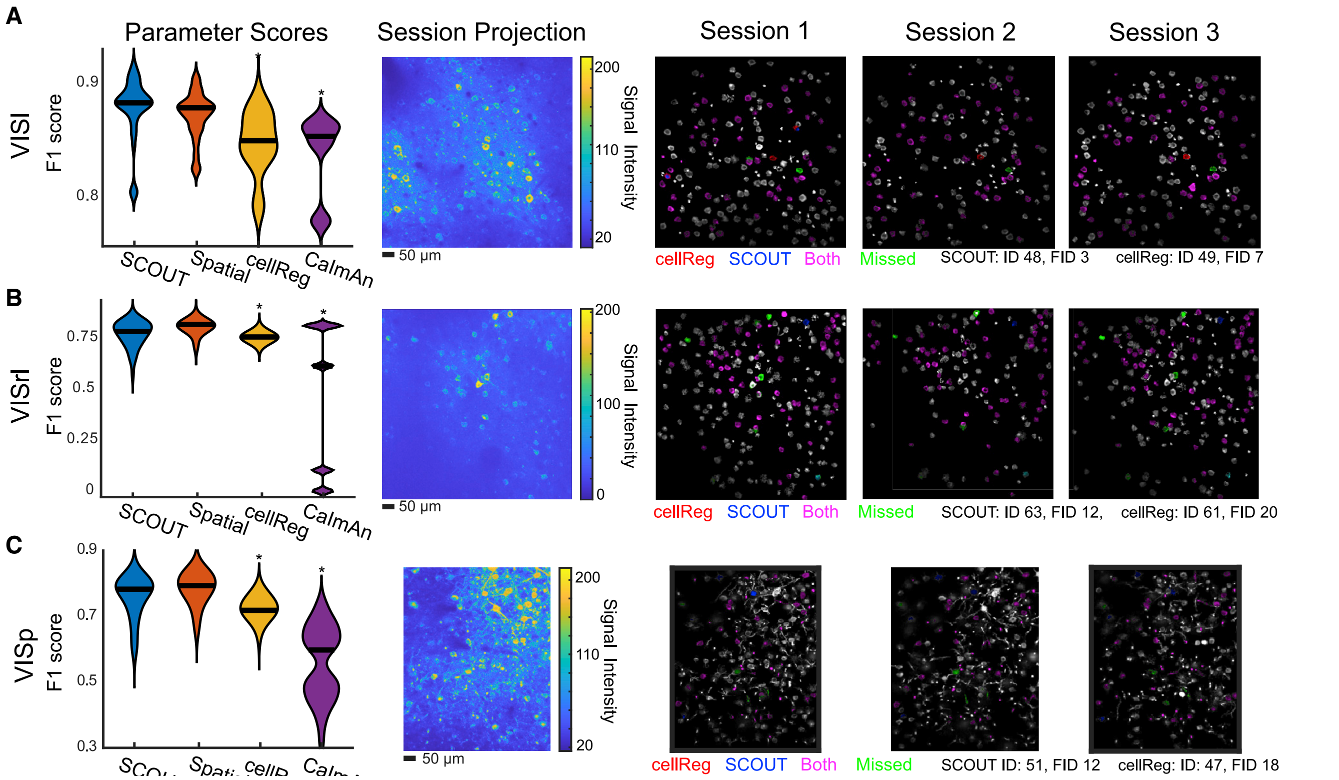


**Figure 5. SCOUT accurately tracks neurons across 2-photon *in vivo* recordings**

(A–C) (Parameter Scores) F1 scores (y axis) computed based on human annotation for *in vivo* 2-photon datasets obtained from the visual cortex (A, VISl; B, VISrl; C, VISp) consisting of three sessions each. Violin plots with medians constructed using F1 scores across parameters after outlier removal and computed using kernel density estimation. Asterisks indicate statistically significant differences compared with SCOUT (ANOVA, Bonferroni). (Session Projection) Max projection of the first session of each recording. (Sessions 1–3) Identified neurons from cellReg and SCOUT overlaid on max projection of the human annotated neurons tracked through all sessions for sessions 1–3 of each recording.

See also Figure S6.

cells (and non-place cells with non-place cells) across sessions (average difference across datasets and thresholds, percentile: 0.150 ± 0.106; percentile threshold range = [0.95, 0.99]; threshold: 0.068 ± 0.070; information score threshold range = [0.5, 1.5]; mean ± SE, Figure 6D), although the threshold result is biased by the third recording.

In summary, neuron pairs identified exclusively by SCOUT exhibit higher average place-field similarity while also exhibiting a larger fraction of pairs with highly similar place fields. Additionally, SCOUT-identified pairs are more likely to lie within the same categorization of either place cells or non-place cells. Although at the specified thresholds SCOUT identified fewer neurons than cellReg, the average JS divergence for SCOUT pairs identified by SCOUT is lower than for cell pairs identified by both methods (in two out of three recordings), while the opposite is true for cellReg (Figure 6B). This indicates the significant possibility of a higher false identification rate for cellReg as is seen in the previous 1-photon datasets.

## Effects of parameter selection on cell-tracking results

Computing mean normalized SD of F1 scores on 1-photon *in vivo* datasets (after outlier removal) gives 0.067 ± 0.0072, 0.066 ± 0.0087, and 0.090 ± 0.0144 (mean ± SE) for SCOUT, cellReg, and CalmAn, respectively. Computing mean normalized SD on the 2-photon datasets gives 0.041 ± 0.0098, 0.032 ± 0.012, and 0.12 ± 0.11 for SCOUT cellReg, and CalmAn, respectively. This suggests comparable parameter stability between SCOUT and cellReg, with somewhat higher variability using CalmAn. Computing the percentage of SCOUT parameters producing higher F1 scores than the maximum produced by CalmAn and cellReg on each dataset gives an average of 99.1% ± 0.9% on the 1-photon dataset and an average of 48.7% ± 20% On the 2-photon datasets.

We compute the ratio of F1 score to maximum F1 score for each method and dataset across all parameters. Higher values indicate results closer to the optimum. Averaging across datasets, we find that SCOUT (median 0.921) exhibited significantly higher ratios than cellReg (median 0.851) and CalmAn (median 0.852) ($p < 1.2 \times 10^{-5}$, Wilcoxon rank-sum test), implying that SCOUT produces highly consistent results across parameters when averaging across datasets. Only 11% of cellReg parameters and 0% of CalmAn parameters produce F1 ratios exceeding the median SCOUT value. We also identify a parameter range (labeled on Figures 7A–7C) that consistently produces strong results (average 0.950 ± 0.0014, F1 ratio to optimal). These parameters emphasize a low threshold for individual identification of

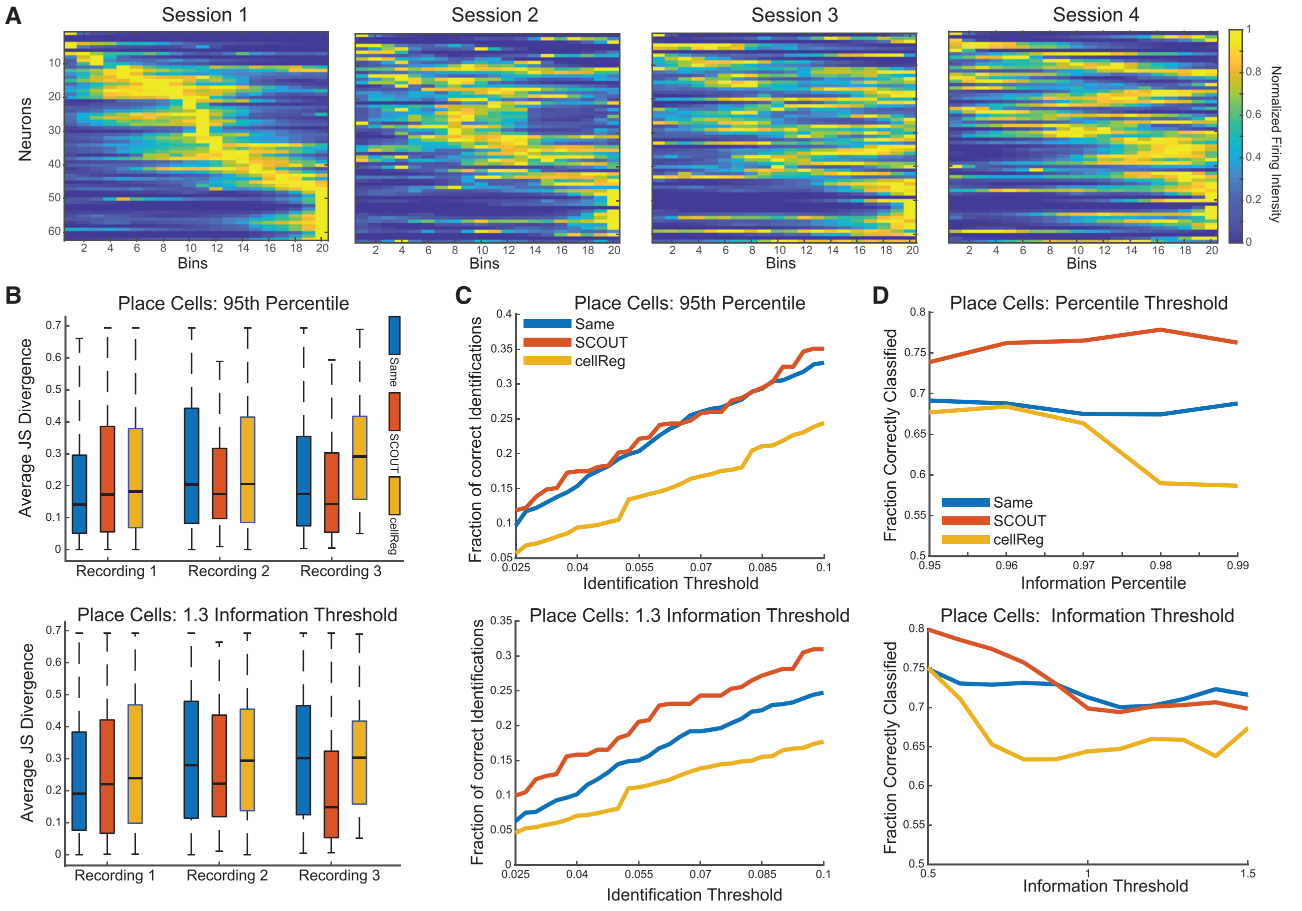


**Figure 6. Testing cell-tracking results using place-field stability**

(A) Sample place fields ordered from left to right (indexed via the first session) after normalization by peak intensity.

(B) Boxplot of JS divergence (y axis) for each recording computed for identified neuron pairs by each method in which either one neuron exceeded the 95$^{th}$ percentile (top) or a hard threshold of 1.3 (bottom).

(C) The weighted average (across recordings) of the fraction of identified cell pairs (y axis) which exhibit pairwise JS divergence lower than a specified threshold (x axis), computed using cell pairs in which one neuron exceeded the 95$^{th}$ percentile (top) or a hard threshold of 1.3 (bottom).

(D) The weighted average (across recordings) of the fraction of identified cell pairs (y axis) which consisted of either both place cells or both non-place cells for information percentile thresholds (top) and information score thresholds (bottom).

neurons across sessions (*min_prob*, range 0.55–0.75), combined with a high threshold for the acceptance of identified clusters (*chain_prob*, 0.75).

In summary, SCOUT exhibits comparable or higher parameter stability compared with other methods while consistently returning top F1 scores. On the 1-photon recordings, virtually every tested parameter choice produces higher F1 scores than competing methods, while nearly 50% of 2-photon results also outperforms the top-line CaImAn and cellReg F1 scores. Finally, averaging results across all datasets, the median F1 score (across parameter choices) exceeds 90% of the maximum, and we identify a parameter range on which average F1 scores consistently exceed 95% of the maximum.

## DISCUSSION

Here we present SCOUT, a novel cell-tracking method applicable to both 1-photon and 2-photon recordings. SCOUT exhibits robust performance on all of the tested datasets, generally exceeding the performance of commonly used methods such as cellReg and CaImAn in simulated situations involving significant spatial/morphology shifts or high noise levels, and *in vivo* recordings in general. SCOUT retains strong performance even in the presence of confounding variables such as non-rigid spatial shifts and poor signal extraction quality by incorporating temporal metrics, a novel clustering algorithm, and consensus clustering.

While SCOUT was initially motivated for use with 1-photon recordings, we also demonstrate robust performance on 2-photon recordings, although the inclusion of temporal metrics here does not significantly improve results on the tested data. The significant difference between 1-photon and 2-photon results are likely due to the stronger signal quality in 2-photon data, which may reduce the discriminatory power of some temporal metrics such as SNR in this context.

SCOUT exhibits strong performance even on recording sessions impacted by experimental conditions. Application of

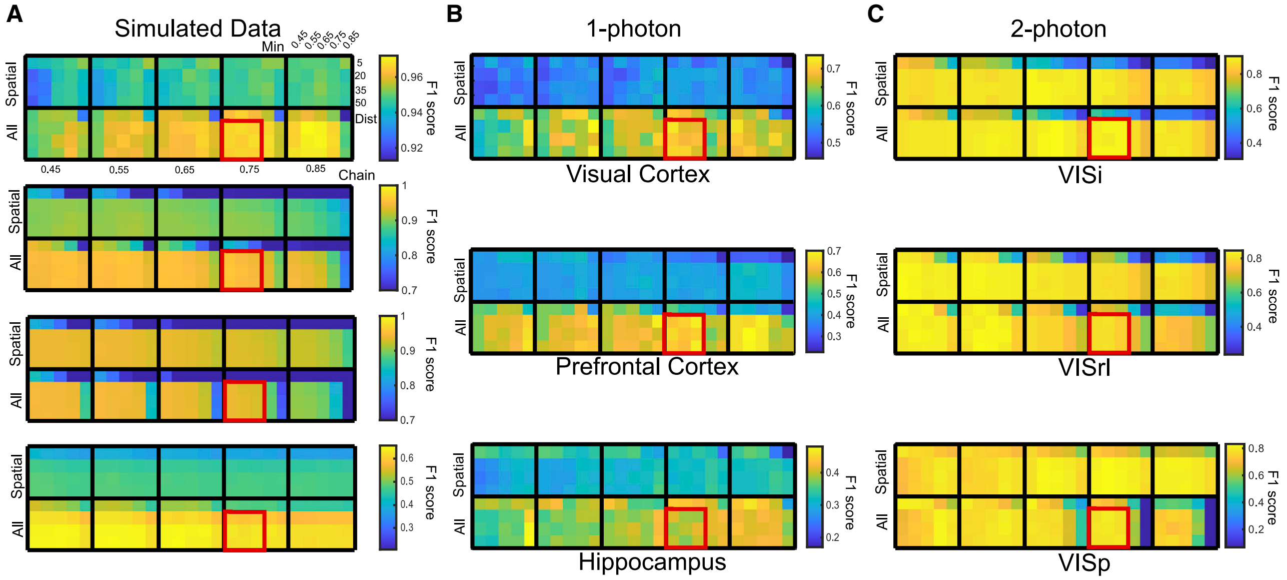


**Figure 7. SCOUT F1 scores by parameter for each dataset**

Heatmaps denote F1 scores across parameters. Each parameter in the "All" rows are obtained using SCOUT with all metrics, while the "Spatial" rows use only spatial metrics. Each box contains vertical parameter changes corresponding to the *max_dist* parameter, and horizontal parameter changes corresponding to the *min_prob* parameter. Movement horizontally across boxes corresponds to the *chain_prob* parameter. Parameter ranges discussed in the text are labeled in red.
(A) SCOUT F1 score averages for the (top to bottom) Gaussian, Non-Rigid 1p, Non-Rigid 2p, and Individual Shift datasets.
(B) SCOUT F1 scores for 1-photon recordings labeled as (top to bottom) visual cortex, prefrontal cortex, and hippocampus.
(C) SCOUT F1 score for 2-photon recordings from the visual cortex labeled as (top to bottom) VISl, VISrl, and VISp.

ketamine, NRG1, or CNO as is present in the 1-photon datasets produces significant impact on the signal intensity of individual neural signals (Grieco et al., 2020; Sun et al., 2019), but inclusion of temporal metrics still significantly improves overall results.

Testing SCOUT on 1-photon hippocampal recordings, we have analyzed cell-tracking output using place-field stability as measured by three separate metrics for which SCOUT produces strong performance. Neuron pairs identified exclusively by SCOUT exhibit lower JS divergence between place fields, while a higher average percentage of SCOUT-identified neuron pairs exhibited consistent place fields and a higher average percentage of SCOUT-identified neuron pairs are of the same type (place cell to place cell or non-place cell to non-place cell) when compared with cellReg.

SCOUT quickly identifies neurons across multiple sessions, with cell tracking taking less than 10 min across up to 30 sessions (depending on the number of neurons). Increasing the number of sessions or neurons per session can significantly increase the runtime, which can be addressed using a combination concatenation-cell-tracking methodology (see STAR Methods) or by thresholding the neuron footprints to reduce spatial overlap, thereby decreasing the component size when clustering.

In conclusion, SCOUT shows strong cell-tracking performance on both simulated and *in vivo* datasets. We have shown that inclusion of temporal metrics when identifying cells across sessions significantly increases the quality of cell tracking. We have also shown that SCOUT exhibits strong parameter consistency over a relatively large parameter range across all datasets. We foresee that the new concepts and techniques used in SCOUT will improve capabilities for long-term cell-tracking-related experiments, particularly in complex situations where SCOUT retains strong performance compared with alternative methods.

## Limitations of the study

While temporal metrics can improve the identification of similar neuron pairs across sessions, strong temporal correlation exhibited between nearby neurons may affect the overall discriminatory power of the temporal correlation metric. In studies involving the functional stability of cells, this may lead to a higher observed stability than true stability, as temporal correlation may lead to the identification of different but functionally similar cells. SCOUT automatically computes the discriminatory power of each metric, which may guide the user in adjusting weight values. In any case, the correlation metric may be disabled (by setting the associated weight to zero).

## STAR★METHODS

Detailed methods are provided in the online version of this paper and include the following:

- KEY RESOURCES TABLE
- RESOURCE AVAILABILITY
  - ○ Lead contact
  - ○ Materials availability
  - ○ Data and code availability

- EXPERIMENTAL MODEL AND SUBJECT DETAILS
  - 1-Photon recordings (visual cortex, prefrontal cortex, hippocampus)
  - 1-Photon hippocampus recordings for place field stability
  - 2-Photon recordings
- METHOD DETAILS
  - Simulated recordings
  - Information score computation and place cell identification
  - Preprocessing recordings
  - Optical recording extraction algorithms
  - Calculation of temporal correlation similarity metric across sessions
  - Spatial similarity measures for calculating neuron similarity across sessions
  - Temporal similarity measures for calculating neuron similarity across sessions
  - Assigning identification probabilities with SCOUT
  - Clustering algorithm
  - Ground truth cell register: simulated data
  - Annotated cell register: *in vivo* data
  - Long-term cell tracking with SCOUT
  - Algorithm parameter settings
- QUANTIFICATION AND STATISTICAL ANALYSIS
  - Metric usage
  - Computation of statistics
  - Statistical specifications by section

## SUPPLEMENTAL INFORMATION

Supplemental information can be found online at https://doi.org/10.1016/j.crmeth.2022.100207.

## ACKNOWLEDGMENTS

This work is supported in part by a BRAIN Initiative grant (NS104897, X.X.) from the US National Institutes of Health, an NSF grant DMS1763272 (Q.N.), and a Simons Foundation grant (594598, Q.N.).

## AUTHOR CONTRIBUTIONS

K.G.J., Q.N., and X.X. conceived this work; K.G.J., H.Z., and S.F.G. performed and analyzed the experiments; K.G.J. implemented algorithms, conducted simulations, and prepared figures; K.G.J., S.J., Q.N., and X.X. wrote and edited the paper.

## DECLARATION OF INTERESTS

The authors declare no competing interests.

Received: August 10, 2021
Revised: January 6, 2022
Accepted: April 8, 2022
Published: April 29, 2022

## REFERENCES

Allen (2016). © 2016 Allen Institute for Brain Science (Allen Brain Observatory). https://registry.opendata.aws/allen-br0ain-observatory/.

Apthorpe, N., Riordan, A., Aguilar, R., Homann, J., Gu, Y., Tank, D., and Seung, H.S. (2016). Automatic neuron detection in calcium imaging data using convolutional networks. In Advances in Neural Information Processing Systems, pp. 3270–3278.

Barbera, G., Liang, B., Zhang, L., Gerfen, C.R., Culurciello, E., Chen, R., Li, Y., and Lin, D.-T. (2016). Spatially compact neural clusters in the dorsal striatum encode locomotion relevant information. Neuron *92*, 202–213.

Bonferroni, C. (1936). Statistical Class Theory and Probability, *8* (Publications of the R Higher Institute of Economic and Commercial Sciences of Florence), pp. 3–62.

Cai, D.J., Aharoni, D., Shuman, T., Shobe, J., Biane, J., Song, W., Wei, B., Veshkini, M., La-Vu, M., Lou, J., et al. (2016). A shared neural ensemble links distinct contextual memories encoded close in time. Nature *534*, 115.

Dunn, J.C. (1973). A fuzzy relative of the ISODATA process and its use in detecting compact well-separated clusters. J. Cybern. *3*, 32–57.

Everitt, B.S. (2014). Finite Mixture Distributions (Wiley StatsRef: Statistics Reference Online).

Flusberg, B.A., Nimmerjahn, A., Cocker, E.D., Mukamel, E.A., Barretto, R.P., Ko, T.H., Burns, L.D., Jung, J.C., and Schnitzer, M.J. (2008). High-speed, miniaturized fluorescence microscopy in freely moving mice. Nat. Methods *5*, 935.

Forsberg, D. (2015). Image-registration (version 1) [software]. https://github.com/fordanic/image-registration.

Ghosh, K.K., Burns, L.D., Cocker, E.D., Nimmerjahn, A., Ziv, Y., El Gamal, A., and Schnitzer, M.J. (2011). Miniaturized integration of a fluorescence microscope. Nat. Methods *8*, 871.

Giovannucci, A., Friedrich, J., Gunn, P., Kalfon, J., Brown, B.L., Koay, S.A., Taxidis, J., Najafi, F., Gauthier, J.L., Zhou, P., and Khakh, B.S. (2019). CaImAn an open source tool for scalable calcium imaging data analysis. Elife *8*, e38173.

Grieco, S.F., Qiao, X., Zheng, X., Liu, Y., Chen, L., Zhang, H., Yu, Z., Gavornik, J., Lai, C., Gandhi, S., et al. (2020). Subanesthetic ketamine reactivates adult cortical plasticity to restore vision from amblyopia. Curr. Biol. *30*, 3591–3603.

Grieco, S.F., Qiao, X., Johnston, K.G., Chen, L., Nelson, R.R., Lai, C., Holmes, T., and Xu, X. (2021). Neuregulin signaling mediates the acute and sustained antidepressant effects of subanesthetic ketamine. Transl. Psychiat. *11*, 1–17.

Grijseels, D.M., Shaw, K., Barry, C., and Hall, C.N. (2021). Choice of method of place cell classification determines the population of cells identified. PLoS Comput. Biol. *17*, e1008835.

Jimenez, J., Goldberg, A., Ordek, G., Luna, V., Su, K., Pena, S., Zweifel, L., Hen, R., and Kheirbek, M. (2016). Subcortical Projection-specific Control of Innate Anxiety and Learned Fear by the Ventral hippocampus. Neuroscience Meeting Planner (Society for Neuroscience).

Kitamura, T., Sun, C., Martin, J., Kitch, L.J., Schnitzer, M.J., and Tonegawa, S. (2015). Entorhinal cortical ocean cells encode specific contexts and drive context specific fear memory. Neuron *87*, 1317–1331.

Klaus, M., Paixao, Z., and Paninski. (2017). The spatiotemporal organization of the striatum encodes action space. Neuron *96*, 949.

Kullback, S. (1997). Information Theory and Statistics (Courier Corporation).

Mukamel, E.A., Nimmerjahn, A., and Schnitzer, M.J. (2009). Automated analysis of cellular signals from large-scale calcium imaging data. Neuron *63*, 747–760.

Pachitariu, M., Stringer, C., and Harris, K.D. (2018). Robustness of spike deconvolution for neuronal calcium imaging. J. Neurosci. *38*, 7976–7985.

Pnevmatikakis, E.A. (2019). Analysis pipelines for calcium imaging data. Curr. Opin. Neurobiol. *55*, 15–21.

Pnevmatikakis, E.A., and Giovannucci, A. (2017). NoRMCorre: an online algorithm for piecewise rigid motion correction of calcium imaging data. J. Neurosci. Methods *291*, 83–94.

Pnevmatikakis, E.A., Soudry, D., Gao, Y., Machado, T.A., Merel, J., Pfau, D., Reardon, T., Mu, Y., Lacefield, C., Yang, W., et al. (2016). Simultaneous denoising, deconvolution, and demixing of calcium imaging data. Neuron *89*, 285–299.

Rubin, A., Geva, N., Sheintuch, L., and Ziv, Y. (2015). Hippocampal ensemble dynamics timestamp events in long-term memory. eLife *4*, e12247.

Schindelin, J., Arganda-Carreras, I., Frise, E., Kaynig, V., Longair, M., Pietzsch, T., Preibisch, S., Rueden, C., Saalfeld, S., Schmid, B., et al. (2012). Fiji: an open source platform for biological-image analysis. Nat. Methods *9*, 676.

Sheintuch, L., Rubin, A., Brande-Eilat, N., Geva, N., Sadeh, N., Pinchasof, O., and Ziv, Y. (2017). Tracking the same neurons across multiple days in ca2+ imaging data. Cell Rep. *21*, 1102–1115.

Smith, S.L., and Häusser, M. (2010). Parallel processing of visual space by neighboring neurons in mouse visual cortex. Nat. Neurosci. *13*, 1144.

Sun, C., Kitamura, T., Yamamoto, J., Martin, J., Pignatelli, M., Kitch, L.J., Schnitzer, M.J., and Tonegawa, S. (2015). Distinct speed dependence of entorhinal island and ocean cells, including respective grid cells. Proc. Natl. Acad. Sci. U S A *112*, 9466–9471.

Sun, Y., Jin, S., Lin, X., Chen, L., Qiao, X., Jiang, L., Zhou, P., Johnston, K., Golshani, P., Nie, Q., et al. (2019). CA1-projecting subiculum neurons facilitate object–place learning. Nat. Neurosci. *54*, 1546–1726.

Yu, K., Ahrens, S., Zhang, X., Schiff, H., Ramakrishnan, C., Fenno, L., Deisseroth, K., Zhao, F., Luo, M.H., Gong, L., and He, M. (2017). The central amygdala controls learning in the lateral amygdala. Nat. Neurosci. *20*, 1680–1685.

Zhou, P., Resendez, S.L., Rodriguez-Romaguera, J., Jimenez, J.C., Neufeld, S.Q., Giovannucci, A., Friedrich, J., Pnevmatikakis, E.A., Stuber, G.D., Hen, R., et al. (2018). Efficient and accurate extraction of *in vivo* calcium signals from microendoscopic video data. eLife *7*, e28728.

Ziv, Y., Burns, L.D., Cocker, E.D., Hamel, E.O., Ghosh, K.K., Kitch, L.J., El Gamal, A., and Schnitzer, M.J. (2013). Long-term dynamics of ca1 hippocampal place codes. Nat. Neurosci. *16*, 264.

Ziv, Y., and Ghosh, K.K. (2015). Miniature microscopes for large-scale imaging of neuronal activity in freely behaving rodents. Curr. Opin. Neurobiol. *32*, 141–147.

# STAR★METHODS

## KEY RESOURCES TABLE

| REAGENT or RESOURCE | SOURCE | IDENTIFIER |
|---|---|---|
| Bacterial and virus strains | | |
| pENN.AAV.CamKII.GCaMP6f.WPRE.SV40(AAV1) | Addgene | RRID:Addgene_100834 |
| Experimental models: oranisms/strains | | |
| C57BL/6 | MODEL-AD center, UC Irvine | |
| Deposited data | | |
| Simulated recordings | This paper | https://doi.org/10.5281/zenodo.6407296, https://doi.org/10.5281/zenodo.6407242 |
| 1-photon hippocampus recordings for place field analysis (3 recordings) | This paper | https://doi.org/10.5281/zenodo.6407203 |
| 1-photon visual cortex recording (1 recording) | (Grieco et al., 2020) | https://doi.org/10.5281/zenodo.6407203 |
| 1-photon prefrontal cortex recording (1 recording) | (Grieco et al., 2021) | https://doi.org/10.5281/zenodo.6407203 |
| 1-photon hippocampus recording (1 recording) | (Sun et al., 2019) | https://doi.org/10.5281/zenodo.6407203 |
| 2-photon visual cortex recordings (3 recordings) | (Allen, 2016) | https://doi.org/10.5281/zenodo.6407203 |
| Software and algorithms | | |
| SCOUT | This Paper | https://github.com/kgj1234/SCOUT, https://doi.org/10.5281/zenodo.6407315 |
| CalmAn | (Giovannucci et al., 2019) | https://github.com/flatironinstitute/CalmAn |
| cellReg | (Sheintuch et al., 2017) | https://github.com/zivlab/CellReg |
| NoRMCorre | (Pnevmatikakis and Giovannucci, 2017) | https://github.com/flatironinstitute/NoRMCorre |
| image-registration | (Forsberg, 2015) | https://github.com/fordanic/image-registration |
| CNMF-E | (Zhou et al., 2018) | https://github.com/zhoupc/CNMF_E |
| Visual Coding: Allen Brain Map | (Allen, 2016) | https://portal.brain-map.org/explore/circuits/visual-coding-2p |
| Miniscope Data Acquisition Software | UCLA | miniscope.org |
| MATLAB | Mathworks | www.mathworks.com |
| Other | | |
| Miniscope | UCLA | miniscope.org |
| GRIN Lens | Edmund Optics | #64-519 |

## RESOURCE AVAILABILITY

### Lead contact

Further information and requests for resources should be directed to and will be fulfilled by the lead contact, Qing Nie (qnie@uci.edu).

### Materials availability

This study did not generate new materials.

### Data and code availability

- Data (analysis and raw recording files) can be accessed at Zenodo (https://doi.org/10.5281/zenodo.6407296, https://doi.org/10.5281/zenodo.6407242, https://doi.org/10.5281/zenodo.6407203).

- Code is publicly available on Github (https://github.com/kgj1234/SCOUT) and at Zenodo (https://doi.org/10.5281/zenodo.6407315).
- Any additional information required to reanalyze the data reported in this paper is available from the lead contact upon request.

## EXPERIMENTAL MODEL AND SUBJECT DETAILS

### 1-Photon recordings (visual cortex, prefrontal cortex, hippocampus)

Recordings were taken from the visual cortex, prefrontal cortex, and hippocampus. The visual cortex recording was subject to the following protocol (Grieco et al., 2020). An initial pair of baseline sessions were taken on consecutive days. After baseline collection on the second day, the animal received a ketamine wash treatment followed by a second recording session. Day 3 consisted of a baseline recording session, followed by NRG1 wash and a second recording session. A single recording session was taken on days 4 and 5. The 7 individual sessions consisted of 4,000–9,000 frames. All tested animals were male.

The prefrontal cortex recording was subject to the following protocol (Grieco et al., 2021). An initial pair of baseline recordings were taken on consecutive days. After baseline collection on the second day, the animal received a ketamine wash treatment followed by a second recording session. Further recordings were taken 2hr, 24hr, 48hr and 72hr after ketamine treatment. The 7 individual sessions consisted of 4,000–9,000 frames.

The hippocampus recording was subject to the following protocol (Sun et al., 2019). Two baseline control recordings taken on consecutive days, were followed on the following day by treatment with CNO (clozapine-N-oxide) and a third session. A post control session was taken after 4 days. Sessions consisted of 7,000–9,000 frames.

Recordings were obtained from and used with permission of the Xu lab (https://sites.uci.edu/xulab/). Recordings previously published in (Grieco et al., 2020; Grieco et al., 2021; Sun et al., 2019).

### 1-Photon hippocampus recordings for place field stability

C57BL/6 mice were obtained from the MODEL-AD center at the University of California, Irvine. Mice were housed under a controlled environment with temperature maintained at 21 - 23°C and humidity at 40%–70%. Mice had free access to water and diet except water restriction during linear track test. The age was 8–10 months at the time of test, both sexes were included since we didn't observe difference of behavior and calcium activity between male and female mice. All the experimental protocols were approved by the IACUC of the University of California, Irvine.

Surgery was performed as described previously (Sun et al., 2019). Briefly, AAV1-CaMKIIa-GCaMP6f-WPRE-SV40 was purchased from Addgene. Mice were anesthetized with 1.5–2% isoflurane and placed on a stereotaxic instrument (Stoelting). Virus was injected into dorsal CA1 (AP -1.94, L 1.4, DV -1.38 mm, relative to bregma) of the right hemisphere using a glass micropipette. The diameter of the pipette tip was 20 - 30 um. The virus titer was $1 \times 10^{13}$ GC/mL and injection volume was 0.3 μL. Mice were treated with carprofen (3 mg/kg) as analgesia for 3 days after surgery.

To record CA1 neurons, a GRIN lens was implanted two weeks after virus injection. Mice were anesthetized with isoflurane, and carprofen and dexamethasone (2 mg/kg) were administered. A 2-mm-diameter cranial window was drilled over CA1, centered at AP -2.3, L 1.75 mm. Then dura was removed with ultrafine forceps, and cortical tissue above the target CA1 area was carefully aspirated using a 29-G blunt needle connected to vacuum, until the vertical striations of corpus callosum appeared. Sterile saline was applied during aspirating. After bleeding was completely ceased, a GRIN lens (1.8 mm diameter, 4.3 mm length, 0.25 PITCH, Edmund Optics) was lowered to contact the corpus callosum (depth −1.55 mm) for CA1 imaging and secured with superglue and dental cement. The skull and lens were covered with Kwik-Sil silicone elastomer (WPI), and mice were allowed to recover for 2–3 weeks.

Mice were anesthetized again, Kwik-Sil was removed and a miniscope (UCLA) mounted onto a baseplate was placed on the GRIN lens to search the imaging area. After cells being in focus, the baseplate was attached on the skull with dental cement, miniscope was removed and a plastic cap was placed on the baseplate to prevent dust.

Mice were water restricted until their bodyweight reached 85–90% of the initial weight, then they were trained to run back and forth on a 1-meter-long linear track to obtain 10–20 μL of water reward on either end of the track. After 5 days of training, miniscope was tethered and mice were trained for another 5 days. The testing consisted of two trials at 30 min apart each day and was repeated for 3 days. On the first day, the linear track was placed in the initial orientation of training. On the second day, the track remained the initial orientation in the first trial and rotated for 90° in the second trial. On the third day, the orientation was 90-degree rotated in the first trial and back to the initial orientation in the second trial. Mice were tested for 30 laps in each trial, usually finishing in 10–15 min. Calcium activity of CA1 neurons was recorded by miniscope, and mouse behavior was recorded by a Logitech webcam simultaneously. Linear track was cleaned with 70% ethanol before each recording. Place field consistency analysis was computed on recordings taken only with the initial orientation, resulting in four sessions.

Mouse IDs (in archived data) are 533 (Female), 536 (Male), and 545 (Female). Data not previously published.

### 2-Photon recordings

2-photon recordings were obtained from the Allen Brain Observatory AWS archive (Allen, 2016). Recordings consisted of three sessions. Recordings were temporally downsampled by a factor of 2, and the first 5,000 frames of each session were used for testing.

Experiments consisted of showing head fixed mice separate visual protocols on three separate days. Protocols consisted of drifting gratings, static gratings, sparse noise, natural scenes and natural movies. See Allen brain observatory documentation for additional information (https://help.brain-map.org/display/observatory/Documentation).

Allen Brain Atlas access information. VISl: Experiment ID 564425775, Genotype: Emx1-IRES-Cre/wt; Camk2a-tTA/wt; Ai93(TITL-GCaMP6f)/wt. VISrl: Experiment ID 660510591, Genotype: Cux2-CreERT2/wt; Camk2a-tTA/wt; Ai93(TITL-GCaMPf)/wt VISp: Experiment ID 642651896, Genotype: Rorb-IRES2-Cre/wt; Camk2a-tTA/wt; Ai93(TITL-GCaMP6f)/wt. Gender not noted in Allen API.

## METHOD DETAILS

### Simulated recordings

For all simulated datasets, neuron footprints were simulated as 2-dimensional Gaussian probability distributions, with diagonal covariance matrices. Spatial footprint width was between 20 and 25 pixels. Spikes were simulated from a Bernoulli distribution with probability of spiking per timebin 0.01, and then convolved with a temporal kernel $g(t) = exp(-t/\tau_d) - exp(-t/\tau_r)$, with fall time $\tau_d = 6$ timebins, and rise time $\tau_r = 1$ timebins. Local background spatial footprints were simulated as 2-D Gaussians, but with larger covariance entries than for the neuron spatial footprint. Blood vessel spatial footprints were simulated using a cubic function, which was convolved with a 2-D Gaussian (Gaussian width: 3 pixels). A random walk model was used to simulate temporal fluctuations of local background and blood vessels. 23 background sources were used throughout all simulated experiments, except for the Individual Shift dataset, in which no background sources were present.

Four sets of recordings were simulated for testing purposes. The Gaussian dataset consisted of 11 recordings with 2000-10,000 frames each, with a 256 x 256-pixel FOV. Each recording was simulated using 50–200 neurons. The Non-Rigid 1-photon dataset consisted of 39 footprint recordings consisting of four sessions of 2000 frames each. Each simulated spatial footprint was transformed with a different individual non-rigid transformation in each session. This transformation was primarily in place, with little translational effect (<2 pixels). The Non-Rigid 2-photon dataset consisted of a copy of the Non-Rigid 1p dataset, in which Gaussian noise was replaced with salt and pepper noise, to portray 2-photon conditions more closely. Spatial footprints were converted into characteristic ring shapes via the following transformation. Pixel intensity values were scaled to lie in the [0,1] interval. All pixels with intensity higher than 0.5 were then replaced by the same intensity subtracted from one. The Individual Shift dataset consisted of 29 recordings consisting of two 3000 frame sessions, with a 100 × 100-pixel FOV. Each recording was simulated using 50–100 neurons. The individual spatial footprints were shifted independently by between 5 and 7 pixels (~30%–40% neuron width) in the second session.

### Information score computation and place cell identification

The method for identifying place cells is based on a previous study (Sun et al., 2019). For each neuron, peak activity locations of neuron activity (neuron.S) were identified, and associated peak intensities were computed. As CNMF-E can produce a significant number of outlying peak intensity values, low outliers were removed by setting a threshold of (0.5)*median(peak intensity), and peak intensities exceeding a threshold equal to three median absolute deviations from the median peak intensity (the MATLAB default) were set to this upper bound. Finally, the output was smoothed using a Gaussian kernel (width 0.5).

Next, we identified time points where the mouse was within 10% of the distance from either end of the linear track, or when mouse speed was lower than an estimated movement threshold, and the corresponding neural signal was removed from the analysis. Finally, we divided the interior region (i.e. the middle 80% of the track) into 20 bins (horizontal axis only) and computed the ratio of the neural signal measured in each bin to the amount of time the mouse spends in each bin to produce the place field.

For each neuron, the mutual information between position and neural activity (information score) was computed as $\sum p_i \lambda_i log2(\lambda_i)$, where $p_i$ denotes the probability of the animal being in each bin, and $\lambda_i$ denotes the ratio of the probability of firing while in the bin to the mean probability of firing. The sum is taken across all bins.

Information percentiles were computed for each neuron by recomputing the information score after randomly shifting the position by at least 3 seconds a total of 500 times. The information percentile was then assigned as the percentile of the non-translated information score in the distribution of translated information scores.

### Preprocessing recordings

*In vivo* recordings were preprocessed using NoRMCorre image registration for motion correction (Pnevmatikakis and Giovannucci, 2017). For experiments taking place over more than one recording, alignment between sessions was performed either manually, by using max projections in imageJ (Schindelin et al., 2012), or automatically using image registration libraries created for Matlab (Forsberg, 2015). SCOUT provides an interface for automatic image registration, as well as manual feature selection-based registration.

When tracking cells on simulated data, no global session registration was performed prior to recording extraction, as no global spatial shifts or deformations were introduced into these datasets. Individual cell tracking methods also had their automatic session registration disabled prior to tracking to remove bias due to global registration method.

### Optical recording extraction algorithms

Calcium imaging extraction produces two outputs from each session: a set of spatial footprints (consisting of pixel intensity values corresponding to each neuron for a given recording session), and the temporal signal (extracted calcium traces; ΔF/F). The spatial

footprints are the primary input for most cell tracking algorithms, while the temporal neural signals are primarily used for downstream analysis.

One class of methods for signal extraction involves semi-manual ROI selection (Pnevmatikakis, 2019). Such methods include manual ROI selection of individual neuron footprints, and subsequent deconvolution of the neural trace, as well as methods such as convolutional neural networks (CNNs) which use a corpus of identified footprints to train a neural network to identify footprints in future experiments (Apthorpe et al., 2016), followed by a second step in which temporal fluorescence traces are extracted based on the proposed footprints. However, such methods become computationally intractable when considering large cell population and become less accurate when considering neurons exhibiting strong spatial overlaps between footprints.

Another class of methods involves automated ROI construction, where both fluorescent traces, and spatial footprints are extracted simultaneously. The simplest such example is PCA/ICA (Mukamel et al., 2009), in which PCA and ICA are successively used to isolate and extract spatial footprints and calcium activity from optical recordings. These methods rely on linear demixing and can produce significant error when neuron footprints exhibit strong spatial overlaps (Pnevmatikakis et al., 2016).

The most recent major advance in 1-photon optical recording extraction (as far as the authors are aware) is CNMF-E (Zhou et al., 2018). As this is the primary method adapted in this paper, we will briefly describe the algorithm.

Given a recording, let $d$ represent the number of pixels in the field of view, $T$ the number of frames observed, and $K$, the number of neurons in the field of view. Then let $Y \in \mathbb{R}_+^{d\times T}$ represent the initial calcium fluorescence recording; let $A \in \mathbb{R}_+^{d\times K}$, the spatial footprints of the neurons, with each column representing the footprint of a single neuron; let the rows of $C \in \mathbb{R}_+^{K\times T}$ represent the fluorescent signal of each neuron at each frame; and let $B \in R_+^{d\times T}$ represent the background fluctuation. The goal is to find $A$,$B$,$C$ such that $\|Y - (AC+B)\|_F$ is minimized, which can be interpreted as determining the optimal spatial footprints, fluorescence traces, and background noise, in order to reconstruct the recording.

The $i$th row of $C$ is represented as an autoregressive process, where $c_i(t) = \sum_{j=1}^{p} \gamma_j^{(i)} c_i(t-j) + s_i(t)$, and $s_i(t)$ represents the number of spikes fired by the $i$-th neuron in the $t$th frame, and $S$, the matrix of spikes, is constrained to be sparse. The footprint matrix $A$ is also constrained to be sparse, and $B$ is constrained to be a nonnegative matrix decomposable as $B = B^f + B^c$ where $B^c$ models the constant baseline background, and $B^f$ models fluctuating background activity. Initialization for neuron centers uses a greedy algorithm, such that a proposed pixel satisfies two criteria: a minimum threshold on peak-to-noise ratio (calculated as peak signal strength divided the standard deviation of the noise), and a sufficiently high temporal local correlation (implying strong similarities in temporal signal for pixels surrounding the proposed center) (Smith and Häusser, 2010). Initialization of variables $C$ and $B$, as well as updates for the background $B$ are discussed in the original paper (Zhou et al., 2018, see also Pachitariu et al., 2018).

Neuron spatial footprints and neural signals for this paper were extracted using CNMF-E.

## Calculation of temporal correlation similarity metric across sessions

Given two preprocessed optical recording sessions $S_1$ and $S_2$, we construct a connecting recording $S_c$ by concatenating the last $n$ frames of the first recording with the first $n$ frames of the second, where $n$ is some number less than the minimum number of frames in $S_1$ and $S_2$. Next, we extract spatial and fluorescence traces from $S_1$, $S_2$, and $S_c$.

Given $N_1$, a neuron from $S_1$, and $N_2$, a neuron from $S_2$, we start by setting a maximal distance threshold $m$, which defines neighboring neurons. If the distance between the centroids $N_1$ and $N_2$ exceeds $m$, $N_1$ and $N_2$ are not considered neighbors. Only neighboring neurons can be identified as the same between sessions. We eliminate from our calculations any neuron in the connecting session exhibiting neural activity in frames overlapping only one of the sessions, as such neurons will not allow comparison between sessions.

For temporal correlation similarity, a similarity score is obtained for each neighboring neuron pair ($N_1$ and $N_2$) in the two recording sessions, by ranging over the full set of neighboring neurons ($N_c$) in the connecting recording (i.e. across the set of $N_c$ coming from $S_c$ such that $N_1$ is a neighbor to $N_c$, and $N_c$ is a neighbor to $N_2$). The choice $N_c$ that maximizes the average of the correlation between $N_1$ and $N_c$, and $N_c$ and $N_2$, is considered the connecting neuron, and the correlation similarity between $N_1$ and $N_2$ is the mean of the maximal correlation across choice of connecting neuron $N_c$ (Figure 1C).

## Spatial similarity measures for calculating neuron similarity across sessions

Currently, three methods for spatial similarity are included with SCOUT: centroid distance, spatial overlap, and Jensen-Shannon divergence. Centroids of neuron spatial footprints are calculated using the usual formulae $\bar{x} = \sum_{i,j} x_i a_{ij}$, $\bar{y} = \sum_{i,j} y_j a_{ij}$, where $i$,$j$ range across the number of pixels in the field of view, in the horizontal and vertical directions respectively, and $a_{ij}$ is the footprint intensity at the $ith$ horizontal pixel, and the $jth$ vertical pixel. Centroid distance between to footprints is calculated as the Euclidean distance between their centroids. Spatial overlap between footprints $a$, $b$ is calculated as $\frac{a \cdot b}{\|a\|_2 \|b\|_2}$, where $a$ and $b$, are binarized column vectors representing whether each footprint has positive pixel intensity. Jensen-Shannon divergence between two (normalized) footprints $P$,$Q$, is calculated as $\frac{1}{2}(D_{KL}(P\|M) + D_{KL}(Q\|M))$, where $M = \frac{1}{2}(P+Q)$, and $D_{KL}$ is the Kullback-Liebler divergence: $D_{KL}(P\|Q) = E(\log[dP/dQ])$, where $dP/dQ$ is the radon-nikodym derivative of $P$ with respect to $Q$.

### Temporal similarity measures for calculating neuron similarity across sessions

In addition to temporal correlation on connecting recordings, several additional temporal similarity measures can be deduced from properties of the fluorescence traces of each neuron. SCOUT has implemented temporal similarities based on signal-to-noise ratio (SNR = Var(Signal)/Var(Noise)), and the fluorescence trace decay rate for each neuron. Signal decay rate is computed automatically in CNMF-E, by fitting exponential models (among other options) to the neural signal. We have provided a similar algorithm for computing signal decay rate directly from the raw neural signal to ensure SCOUT works with other pipelines. This algorithm identifies peak locations, normalizes the signal height at each peak, and fits an exponential decay function to the average signal. This function can fail if fewer than three peaks were detected for a given neuron.

For SNR, similarity between neurons is calculated as $abs(\log(SNR_1) - \log(SNR_2))$ where $SNR_i$ is the signal-to-noise ratio for neuron $i$ (taking the logarithm produces a more centralized distribution of values). Signal decay similarity is calculated as $abs(dec_1 - dec_2)$, where $dec_i$ is the signal decay rate for neuron $i$.

### Assigning identification probabilities with SCOUT

To assign probability scores between sessions for a given metric, we detail two approaches. First, we can simply assign the percentile as the probability score for each metric. If the distance between $N_1$ and $N_2$ for a given a metric, is less than $p$% of distances between all possible neighbor pairs, then $p$ is the percentile assigned to the pairing. This method has several drawbacks. First, it is sensitive to the choice of maximum distance parameter. If the parameter governing the maximum distance between neighbors is increased, the probability assigned to any neighboring pair will increase. Second, when few neuron pairs exist, similarity metric values can accumulate near 0, so that even relatively small metric values can be associated to low probabilities.

Another paradigm is to assume that for each metric, the distances between neighboring pairs come from a mixture of distributions: a distribution of distances corresponding the neurons that should be identified between sessions, and a set of neighbors that are distinct ((Sheintuch et al., 2017)). Before fitting the mixture of distributions, a probability density function is constructed, by applying kernel density estimation to the normalized histogram of distances, using reflected boundaries near theoretical maximum and minimum values (such as 0 or 1 for correlation metrics). Next, we construct a model consisting of the weighted sum of two probability distribution functions, which is then fit to the approximated pdf, using nonlinear regression (Matlab nlinfit).

Given a mixture model consisting of a weight $w$, a model for identified neurons between sessions, $f$, and a model for unidentified neurons between sessions, $g$, the mixture model approximates the probability distribution function $h$, obtained via kernel density estimation from the initial distribution of distances, as $h(x) = wf(x) + (1 - w)g(x)$. Given a proposed distance $x$, the probability that $x$ is sampled from the distribution with pdf $f$, is given by $\frac{wf(x)}{wf(x) + (1 - w)g(x)}$, using Bayes theorem. We have primarily used Gaussian mixture models (Everitt, 2014).

We can also apply soft K-means clustering (Dunn, 1973), an adaptation of K-means in which data points are assigned identification probabilities for each cluster, and a "fuzzifier" is introduced to govern the spread of identifications probabilities, adjusting the crispness of the clusters. Similarly to mixture models, this algorithm separates similarities into identified and non-identified categories, with associated probabilities (Figure 1E (1)). This algorithm frequently identified the most neurons, but with a higher false discovery rate. This is the default algorithm for SCOUT.

Generally, little difference is seen between results with soft K-means and mixture models, except for recordings with only two sessions, for which Gaussian mixture models typically exhibit stronger results. GMM distribution fitting is typically slower than K-means and produces a sharper decision boundary. We recommend using K-means except in the case where the recording consists of only two sessions.

### Clustering algorithm

After computation of temporal metric similarity for all neuron pairs in each pair of sessions, we assign identification probabilities for each metric using a probabilistic model (i.e., soft K-means, GMM). To combine the metric identification probabilities into a single identification probability, we use a weight vector (a 1 × n vector where n is the number of metrics, such that the sum of entries is 1) which governs the emphasis each metric receives. Multiplying this weight vector by the associated identification probabilities for each vector and summing the result results in a single identification probability for each pair of cells in each pair of sessions. These probabilities are placed in a similarity matrix of size n_cells x n_cells, where n_cells is the total number of neurons extracted across all sessions and entries are the identification probabilities between cell pairs. Cells in the same session are assigned a low similarity (−10,000) to prevent clusters from containing more than one neuron from the same session.

This similarity matrix is decomposed into connected components, and each component is clustered according to the following algorithm. Clusters are initialized by placing the least similar neurons in separate clusters. Remaining neurons are added to each cluster based on which addition decreases average cluster inter-neuron similarity the least. If no option is available that keeps the average cluster similarity above the user provided *chain_prob* threshold, a new cluster is created. The process continues until all neurons are assigned a cluster. Similarities between neurons in the same session are set to some high magnitude negative number, to prevent assignment to the same cluster.

At each iteration, we calculate the total increase in average similarity between cluster members (over all clusters) gained by assigning each neuron to a new cluster (a switch) (Figure 1D). We also calculate the total increase in average similarity between

cluster members derived by swapping the cluster assignments of each pair of neurons (a swap). The operation that maximizes the similarity increase is chosen, and the algorithm continues until a maximum number of iterations is reached, or no further gains can be made by these operations. Finally, the minimal average cluster similarity (across clusters) is compared with the *chain_prob* threshold. If the cluster similarity falls beneath this value, a new cluster is created and populated with the least similar neuron in the lowest scoring cluster (ranked by average within cluster similarity), and the process repeats until convergence.

To correct the propensity toward the creation of medium sized clusters (and thus against tracking neurons through all sessions), we add a bias term to the switch and swap scores as follows. First, a switch that increases the maximal cluster size of the clusters involved is rewarded with the addition of a constant bias term, while the reverse is penalized by the subtraction of the bias term. A swap is penalized via subtraction of a bias term if the swap causes the cluster with the larger size to decrease its average identification probability, and vice versa if the cluster with the larger size were to increase the average identification probability. Swaps and shifts that decrease the size of a cluster with inter-neuron similarity exceeding the *chain_prob* threshold are ignored. By placing a limit on the number of times any individual neuron can be swapped or shifted between groups, the algorithm converges in most instances, and usually within a few iterations (<25).

Discrepancies between clustering results due to initial clustering assignments, as well as the problematic usage of a single weight variable for aggregating identification probabilities motivates a consensus clustering framework. We generate random perturbations of the weight vector by adding a random value produced by from an N(0,0.12) distribution (0.12 is $^3/_4$ the individual weight if all metrics are used). Weight values below zero are set to zero, and the vector is renormalized. In the implementation, 29 perturbations are generated (resulting in 30 vectors), which are then used to create similarity matrices which are clustered via the previously described algorithm, but using the connected components defined by the initial weight vector.

Next, we construct a consensus matrix for each component of size n_cells x n_cells, where n_cells is the number of cells in the component, and the entry is the percentage of instances in which the associated cell pair were placed in the same cluster. This consensus matrix was then clustered using the same algorithm described above to produce the final cell register.

## Ground truth cell register: simulated data

As SCOUT requires extracted data both from individual and connecting sessions, we cannot directly use the ground truth cell register. Instead, we use the session extractions to construct a cell register as follows. After dividing each recording into sessions, for each session, if a neuron extracted from that session had spatial correlation greater than 0.65, and temporal correlation greater than 0.8 with the neural signal of a ground truth neuron over the correct frames, the extracted neuron was identified with the ground truth neuron. From this we create the ground truth cell register consisting of identified neurons across all sessions.

## Annotated cell register: *in vivo* data

A human annotated ground truth was determined as follows 1) Neurons identified with visibly recognizable common features on the correlation image of each session are identified; 2) The identified neurons were visually checked, and false identifications were eliminated; 3) If multiple identifications in a single session are still available, all such identified neurons with SNR less than 2 were removed from the ground truth cell register. While filtering neurons with SNR less than 2 may bias the result toward SCOUT, neurons with high SNR showed greater variance in SNR magnitude between sessions than did low SNR neurons, which may reduce the power of this metric.

## Long-term cell tracking with SCOUT

For long term cell tracking, we propose a combination of concatenation and cell tracking. In this methodology, recordings are concatenated into batches of uniform length, with overlapping portions of each batch used to calculate spatiotemporal similarity. This method decreases the number of connecting recordings required but requires spatial footprint stability over each batch.

## Algorithm parameter settings

CNMF-E parameters were set as min_pnr = 5, min_corr = 0.3 merge_thr = [0.65,.7,-1] on *in vivo* recordings), and dmin = [1.5, 15]. All other parameters were left as defaults.

SCOUT parameters were left as defaults, except for *min_prob*: the minimum cell-cell similarity probability for identification, *chain_prob*: the minimum inter-cluster similarity (measured as average cell-cell similarity between all cells in the cluster), *max_dist* (maximal distance between identified cells), and *weights*: the baseline weight each metric is assigned when computing similarity probabilities. The variables *min_prob* and *chain_prob* were assigned values of [0.45,.0.55,.0.65,0.75,0.85], *max_dist* took values [5,20,35,50] (except for the Individual Shift dataset which had one additional increment of 15), and *weights* took values [1/6,1/6,1/6,1/6,1/6,1/6] corresponding to correlation, footprint centroid distance, footprint overlap, footprint KL divergence, signal SNR, and signal decay respectively, except for the Individual Shift dataset tests where the SNR metric was dropped, as this dataset contained no simulated noise. SCOUT without spatial metrics had identical weights for the spatial metrics (centroid distance, overlap, KL divergence), with zeros for the temporal metrics ([0,1/3,1/3,1/3,0,0]). On the 2-photon *in vivo* data, temporal metrics were set at 1/3 the value of the spatial metrics. When computing results for individual temporal metrics, weights were set equally for all used metrics, with the rest left as zeros (i.e. [1/4,1/4,1/4,1/4,0,0] for the correlation metric tests). Computing results for each distinct choice of metrics yielded 500 results per dataset, or 100 per weight choice.

For cell tracking via cellReg on the simulated recordings, we varied *p_same_threshold* across the range [0.2,0.4,0.5,0.6,0.8], and let *maximal_distance* (maximal distance between neighbors) vary between 10 and 50 by increments of 5. For cell tracking via CaImAn, we varied the *max_dist* parameter from 5 to 45 by multiples of 5, and *threshold* from 0.4 to 0.9 by multiples of 0.1.

On the hippocampal place field datasets, we set all metric weights equal for SCOUT, and used a *min_prob* and *chain_prob* threshold of 0.75, with *maximal_distance* parameter 20 (pixels). For cellReg we used a *p_same_threshold* value of 0.6, and a *maximal_distance* parameter of 15 (pixels). These were determined via the best average parameters across the 1-photon data obtained in the section *SCOUT successfully tracks cells on in vivo multi-session recordings*.

## QUANTIFICATION AND STATISTICAL ANALYSIS

### Metric usage

Primary metrics used in this paper are F1 score, Jaccard similarity, and Jensen-Shannon divergence. The F1 metric is defined as $\frac{2 \times PDR \times (1 - FDR)}{(1 - FDR + PDR)}$, where PDR is defined as the percentage of available neurons tracked by a method and FDR is defined as the percentage of tracked neurons containing at least one false identification. Jaccard similarity metric is computed as $|A \cap B| / |A \cup B|$, where $A$ and $B$ represent sets of identified neurons. If more than one neuron is acceptable in the annotated/ground truth cell register, then Jaccard similarity discards the extraneous neuron before computing the final value. Jensen-Shannon divergence is defined as $\frac{1}{2}$ $(D_{KL}(P||M) + D_{KL}(Q||M))$, where $M = \frac{1}{2}(P + Q)$, and $D_{KL}$ is the Kullback-Liebler divergence: $D_{KL}(P||Q) = E(\log[dP/dQ])$, where $dP/dQ$ is the radon-nikodym derivative of $P$ with respect to $Q$.

### Computation of statistics

All statistical tests were performed using *MATLAB* built in functions (namely *ANOVA1*, *ttest*, *ttest2*, *multcompare*, *fitlme*, *ranksum*). Unless stated otherwise, results in text are supplied as mean +/− std. error. Statistical significance was defined with a p value of 0.05. Statistical tests are labeled for each initial comparison. Repeated uses are not labeled. Associated statistical tests are labeled in each figure.

### Statistical specifications by section

In Usage of temporal metrics improve discrimination between identified and non-identified neurons across sessions section, all statistical tests were performed using Wilcoxon rank sum test. Tests were performed individually for each recording. Total neurons in first session: (262, 138, 293 for VC, PFC, Hipp. respectively), (191, 220, 288 for VISl, VISrl, VISp respectively). Median ratios were computed as the ratio of the median values for identified and nearest neurons.

In Testing SCOUT on simulated multi-session recordings, ANOVA followed by Bonferroni multiple comparison test correction was used for identification of significant differences between methods in all cases. Datapoints used are the maximal F1/Jaccard score/ JS_divergence/abs(1-tracking_ratio) for each recording, grouped by methods. This consists of 11 datapoints per method on the Gaussian dataset, 39 datapoints per method on the Non-Rigid datasets, and 29 datapoints per method on the Individual Shift dataset.

In *SCOUT successfully tracks cells on in vivo multi-session recordings*, statistics reported in figures (Figures 4 and 5) test the distribution of results across parameters between methods for each recording using ANOVA and Bonferroni pairwise comparisons after elimination of outlying results from the distribution.

In Testing performance via place cell stability analysis, the primary statistical test was a Linear Mixed Effects model with *MATLAB* equation $consistency \sim method + (method|recording)$, tested on neuron pairs identified exclusively by either SCOUT or cellReg after discarding neurons tracked through only two sessions. This results in an average of 558 ± 208 pairs identified by both methods, 327 ± 182 pairs identified exclusively by cellReg, and 122 ± 48 pairs identified exclusively by SCOUT. Additionally a two-sample t-test is used to validate place field stability (in comparison with all possible identifications).

In Effects of parameter selection on cell tracking results, we test the median F1 scores for each method after averaging across all datasets using a Wilcoxon rank sum test. Data points correspond to the number of parameters tested for each method (100 for SCOUT, 45 for cellReg and CaImAn).

**Cell Reports Methods, Volume *2***

## Supplemental information

# Tracking longitudinal population dynamics of single neuronal calcium signal using SCOUT

**Kevin G. Johnston, Steven F. Grieco, Hai Zhang, Suoqin Jin, Xiangmin Xu, and Qing Nie**

# Supplemental Figure 1

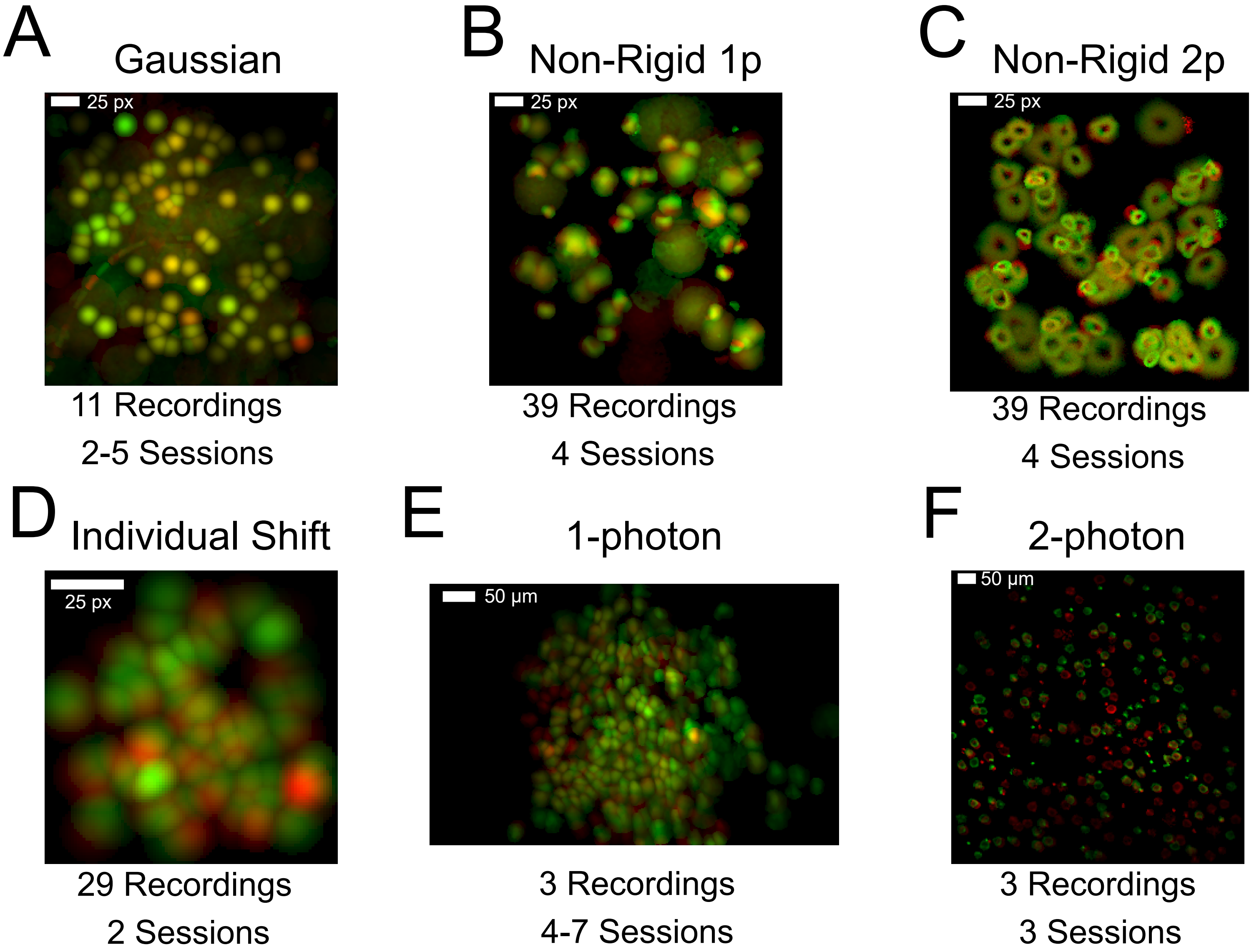

**Supplemental Figure 1**: **Overview of tested data, Related to Figures 3-5**. Neuron extraction from two sessions from each dataset, overlaid to visualize the identification of neurons across each session, as well as the number of recordings and sessions per recording in each dataset. Gaussian (**A**), Non-Rigid 1p (**B**), Non-Rigid 2p (**C**), and Individual Shift (**D**) datasets are simulated, while 1-photon (**E**) and 2-photon (**F**) datasets are *in vivo* recordings. Simulated datasets exhibit different features and difficulties for cell identification across sessions, such as noise (particularly in the Non-Rigid 1p dataset), cell body transformations (Non-Rigid 1p and Non-rigid 2p) and neuron translations (Individual Shift) represent common issues with *in vivo* recordings. Similar effects can be viewed in the *in vivo* recording representations.

# Supplemental Figure 2

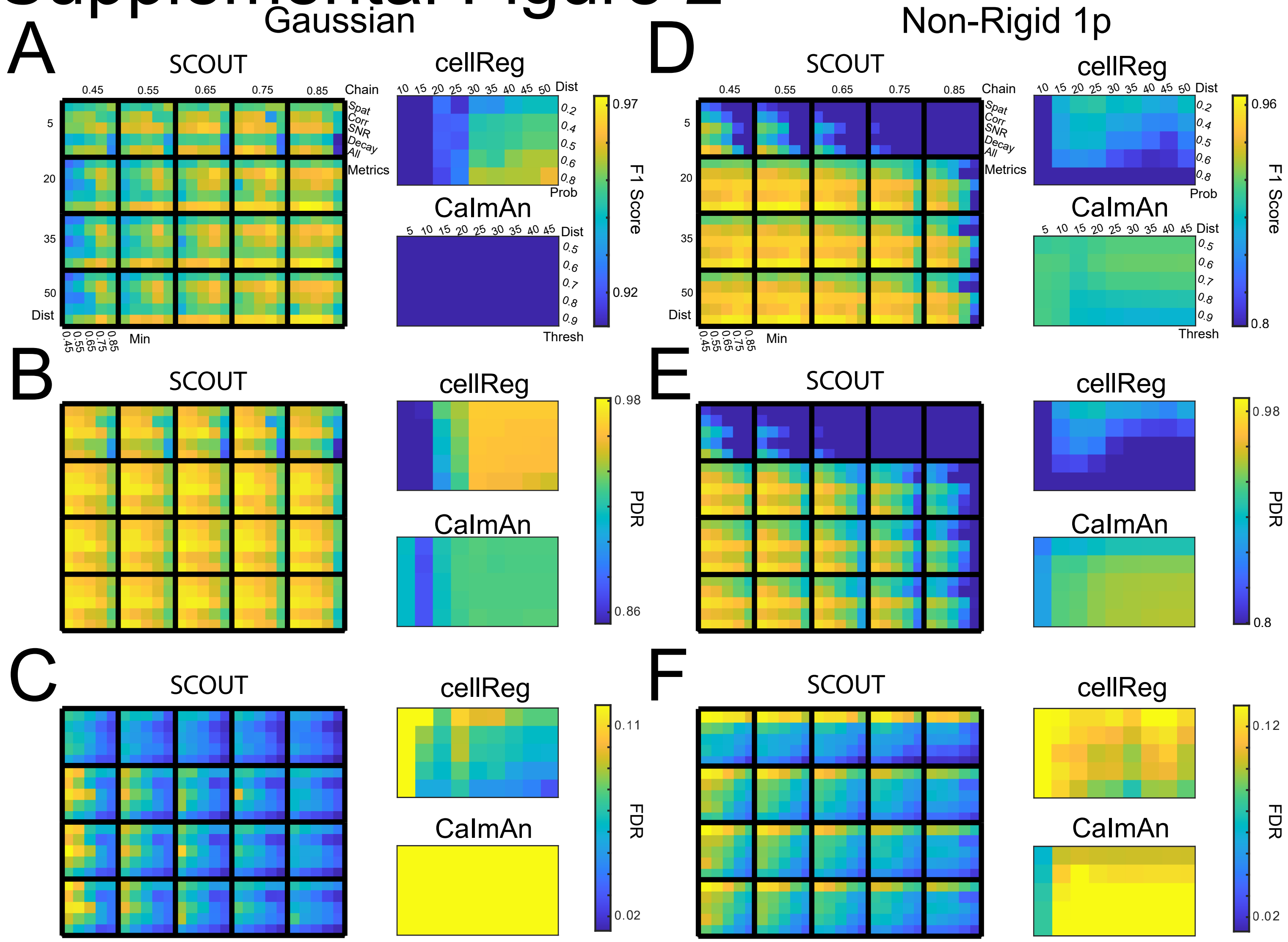

**Supplemental Figure 2**: **Comparison of F1, PDR and FDR scores on the Gaussian and Non-Rigid 1p datasets, Related to Figure 3. A-E**: Each heatmap depicts a different metric for determining cell tracking quality: PDR  the percentage of tracked neurons through all recordings of those present in each recording; FDR the percentage of tracked neurons containing at least one identification error; F1 Score defined as $F1 = 2 * PDR * (1 - FDR)/(1 - FDR + PDR)$. Parameters are labeled in **A(D)** and extend to **B-C(E-F)**. Parameters for SCOUT include *max_dist* (labeled Dist), *min_prob* (labeled min), and *chain_prob* (labeled chain). The vertical change in each box corresponds to which metrics were used in the computation (spatial only, correlation, SNR, decay, or all metrics), the horizontal change in each box corresponds to variation in the *min_prob* parameter. Vertical change across boxes corresponds to variation in the *max_dist* parameter, and horizontal changes across boxes correspond to the *chain_prob* parameter. CaImAn and cellReg have a common maximum centroid distance (Dist) parameter, as well as threshold parameters (Prob, Thresh) governing acceptance levels for tracked cells. Results for Gaussian dataset on left (**A-C**), results for Non-Rigid 1p dataset on right (**D-F**).

# Supplemental Figure 3

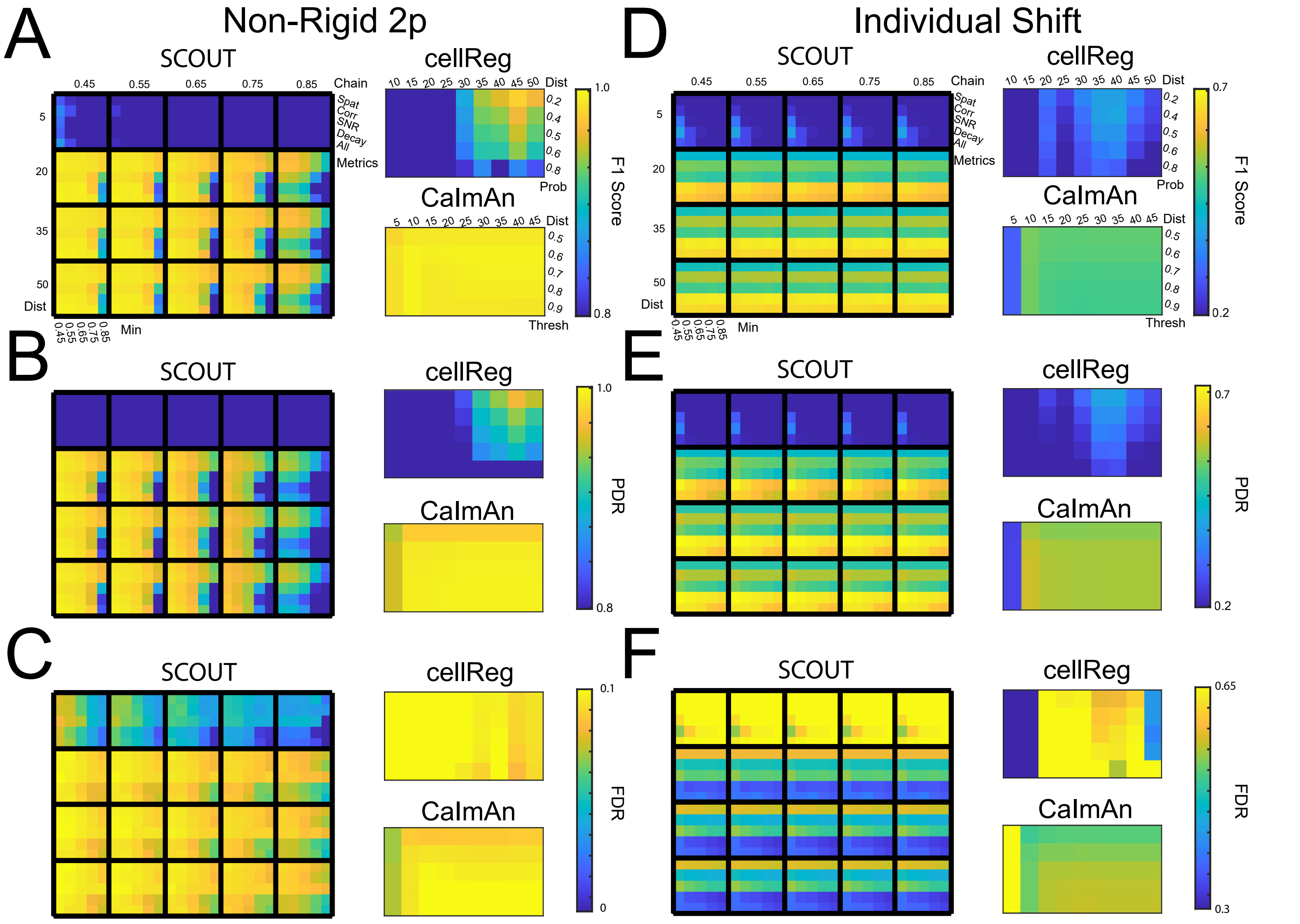

**Supplemental Figure 3**: **Comparison of F1, PDR, and FDR scores on the Non-Rigid 2p and Individual Shift datasets, Related to Figure 3.** **A-C**: F1 scores (**A**), PDR (**B**), and FDR (**C**) for SCOUT, cellReg, and CaImAn across a variety of parameters for the Non-Rigid 1p dataset. Parameter labels (**A**) extend to **B-C**. **D-F**: F1 scores (**D**), PDR (**E**), and FDR (**F**) for SCOUT, cellReg, and CaImAn across a variety of parameters for the Non-Rigid 1p dataset. Parameter labels (**D**) extend to **E-F**. Results for the Non-Rigid 2p dataset on the left (**A-C**), results for the Individual Shift dataset on the right (**D-F**).

# Supplemental Figure 4

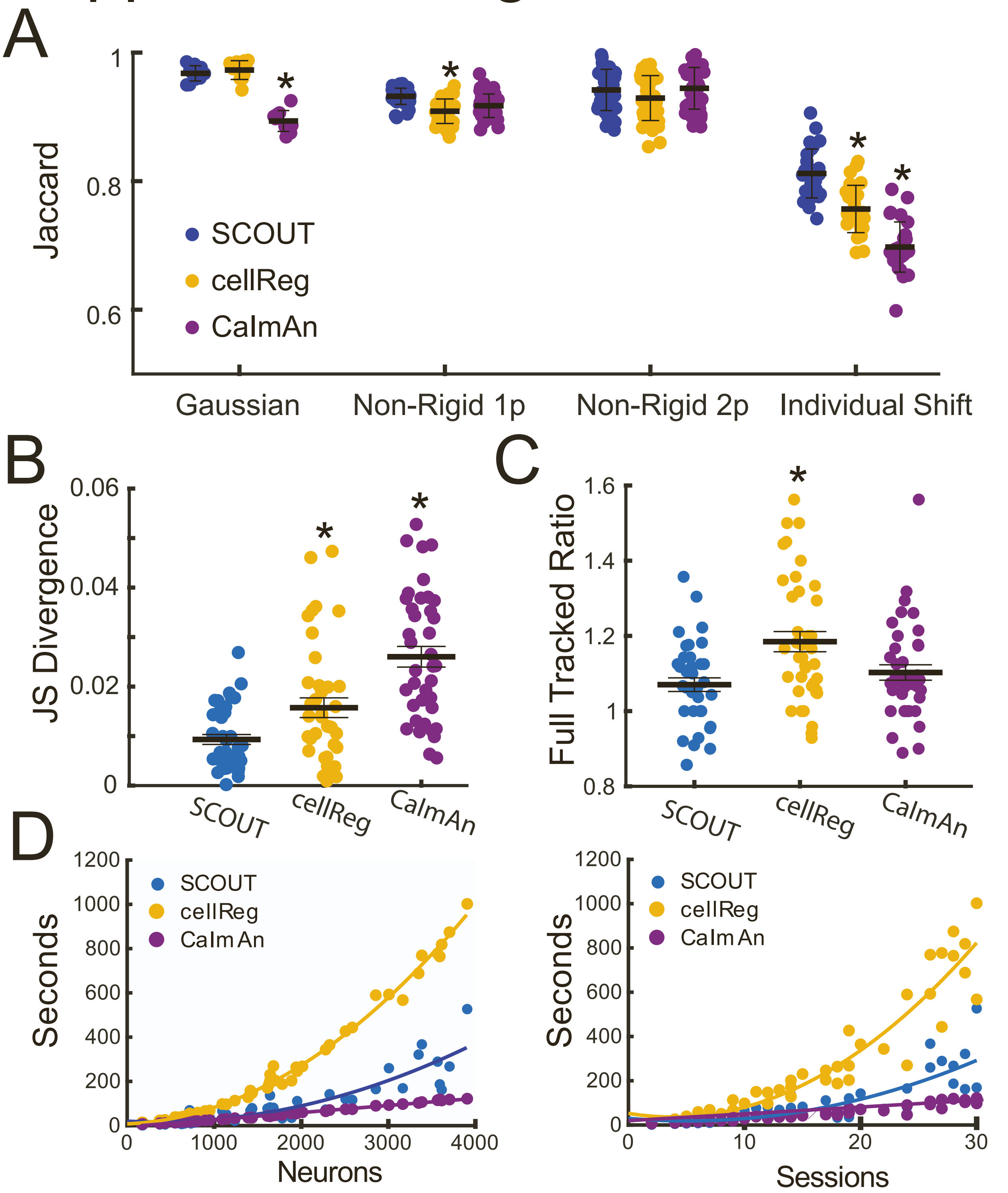

**Supplemental Figure 4: Supplemental statistical comparisons of methods, Related to Figure 3. A:** Maximal Jaccard similarity (y-axis) on modified versions of the simulated datasets (x-axis), in which 30% of neurons were removed from analysis prior to cell tracking. **B:** JS divergence (y-axis) between cluster size distributions of inferred cell registers for each method (x-axis), on the Non-Rigid 1p dataset after removal of 30% of neurons, based on parameters producing highest F1 scores. **C:** Full tracking ratios (y-axis, the percentage of available neurons tracked through all sessions) on the modified Non-Rigid 1p dataset based on parameters producing highest F1 scores. **D:** Algorithm runtime comparison between SCOUT, cellReg and CaImAn. Testing was performed on the Non-Rigid 1p dataset. We performed 50 tests in the following manner: 1) a random recording was identified; 2) recording sessions were duplicated to construct up to 30 sessions; 3) SCOUT, cellReg and CaImAn were run with global session registration disabled, and without the JS metric (SCOUT only) to provide the most unbiased results. Tests were run with 24 cores on a 2.2 Ghz CPU. Results are shown based on the seconds required to complete cell tracking (y-axis), and the total number of neurons (left) or sessions (right) in the recording. SCOUT and cellReg results were fit with a quadratic curve, and CaImAn with a linear curve. For panels **A-C**, statistical results denote pairwise comparison with SCOUT and are labeled with black asterisks (ANOVA, Bonferroni multiple comparisons). Black bars indicate mean ± SE.

# Supplemental Figure 5

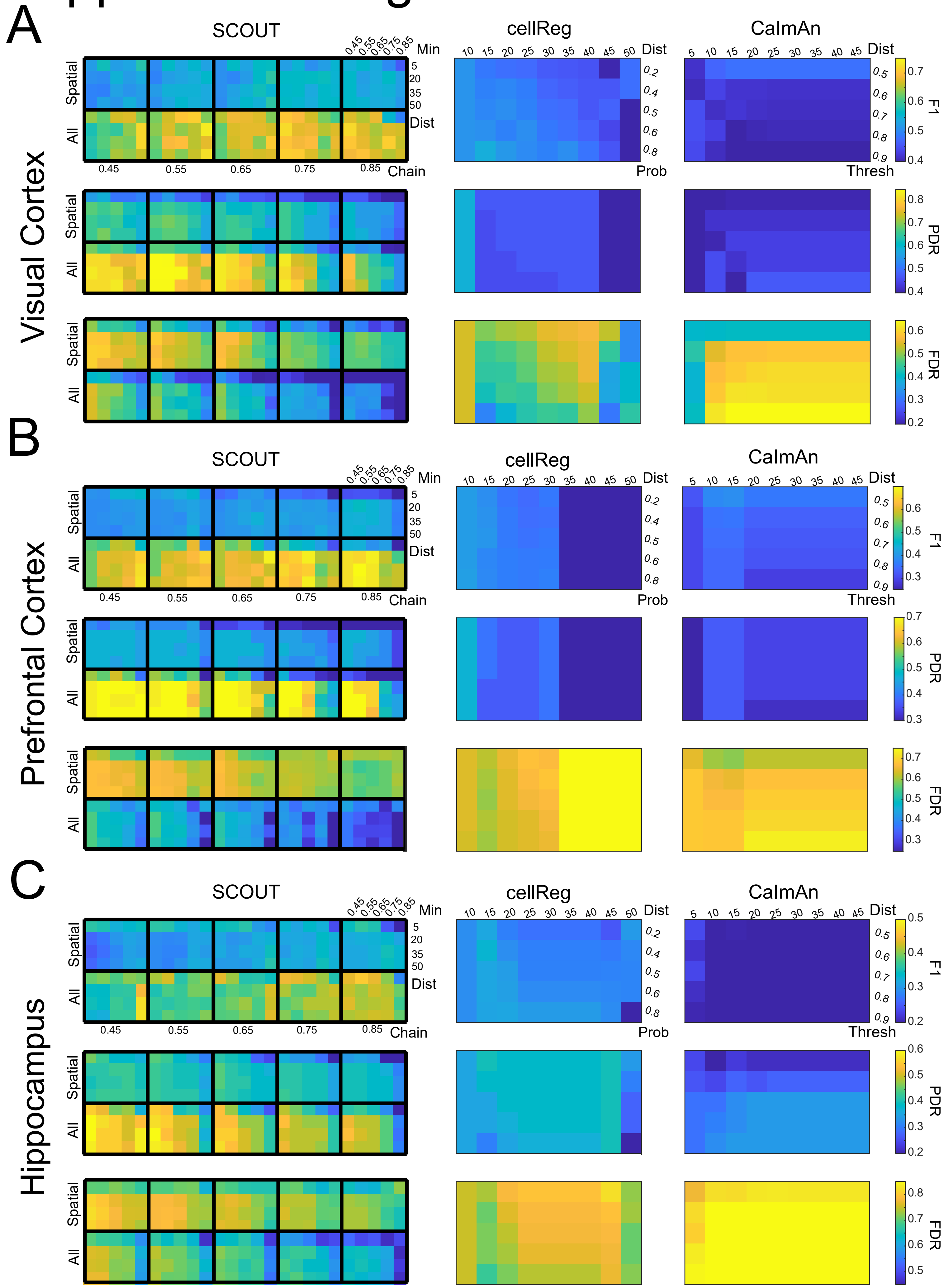

**Supplemental Figure 5**: **SCOUT cell tracking performance on 1-photon *in vivo* recordings, Related to Figure 4. A**: F1 scores (top), PDR (middle), and FDR (bottom) for SCOUT, cellReg, and CaImAn across a variety of parameters (labeled top), for the visual cortex recording. **B**: F1 scores (top), PDR (middle), and FDR (bottom) for SCOUT, cellReg, and CaImAn across a variety of parameters (labeled top) for the prefrontal cortex recording. Multiple cellReg parameters resulted in errors, the associated F1 and PDR were set to 0, and FDR to 1. **C**: F1 scores (top), PDR (middle), and FDR (bottom) for SCOUT, cellReg, and CaImAn across a variety of parameters (labeled top) for the hippocampus recording.

# Supplemental Figure 6

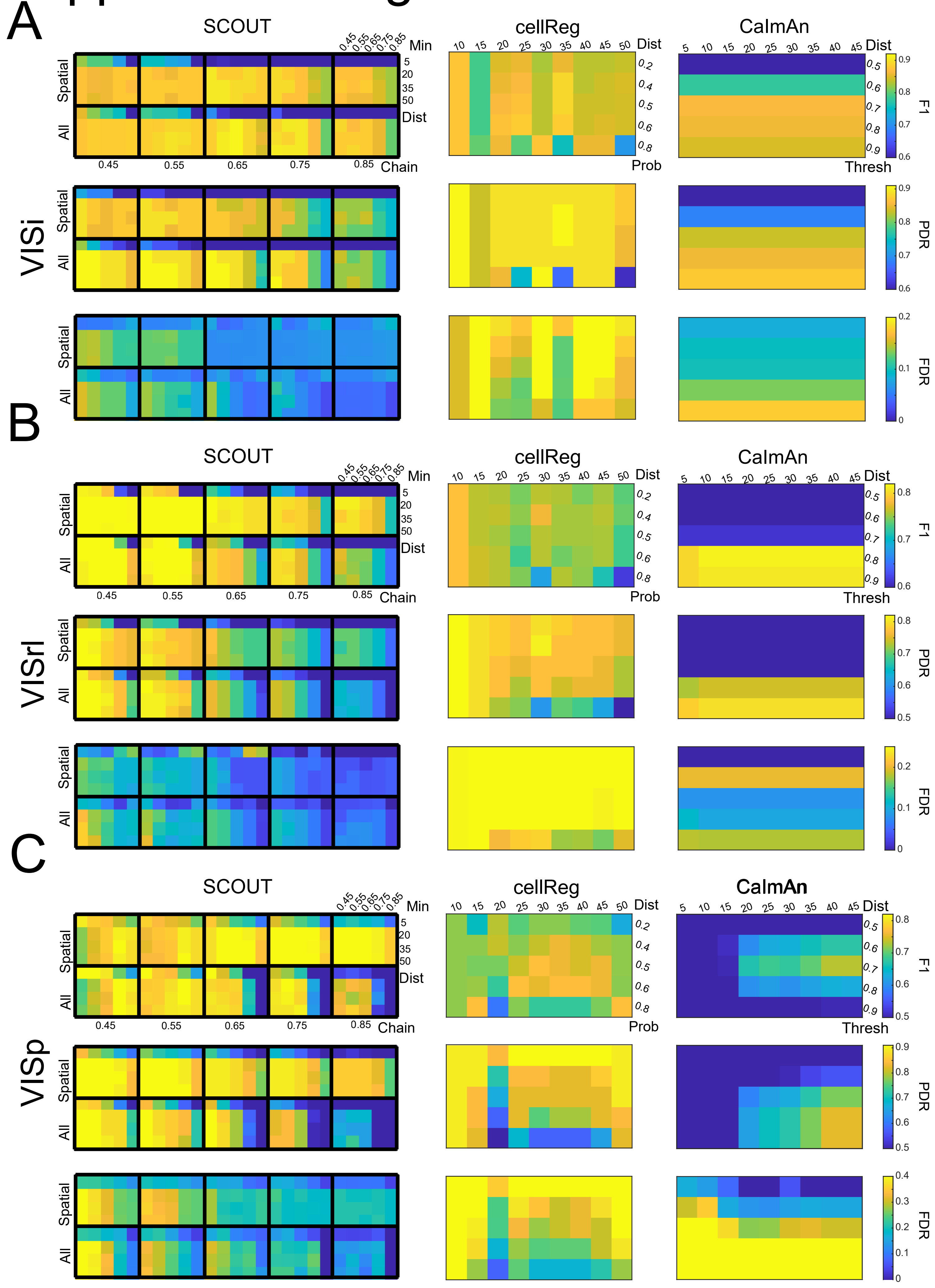

**Supplemental Figure 6**: **SCOUT cell tracking performance on the 2-photon *in vivo* recordings, Related to Figure 5**. **A**: F1 scores (top), PDR (middle), and FDR (bottom) for SCOUT, cellReg, and CaImAn across a variety of parameters (labeled top) for the VISi recording. **B**: F1 scores (top), PDR (middle), and FDR (bottom) for SCOUT, cellReg, and CaImAn across a variety of parameters (labeled top) for the VISrl recording. **C**: F1 scores (top), PDR (middle), and FDR (bottom) for SCOUT, cellReg, and CaImAn across a variety of parameters (labeled top) for the VISp recording.

# Supplemental Table 1

| | | *Gaussian* | *Non-Rigid 1p* | *Non-Rigid 2p* | *Ind. Shift* |
|---|---|---|---|---|---|
| ***SCOUT*** | F1 Score | *0.986 +/- 0.005* | ***0.970 +/- 0.003*** | 0.956 +/- 0.005 | ***0.707 +/- 0.013*** |
| ***Spatial*** | F1 Score | *0.967 +/- 0.007* | 0.929 +/- 0.005 | 0.949 +/- 0.005 | 0.526 +/- 0.010 |
| ***cellReg*** | F1 Score | *0.964 +/- 0.007* | 0.916 +/- 0.005 | 0.938 +/- 0.006 | 0.500 +/- 0.016 |
| ***CalmAn*** | F1 Score | 0.876 +/- .0131 | 0.924 +/- 0.004 | 0.953 +/- 0.006 | 0.547 +/- 0.015 |
| ***SCOUT*** | Jaccard | *0.97 +/- 0.004* | ***0.93 +/- 0.002*** | 0.93 +/-0.006 | ***0.78 +/- 0.011*** |
| ***cellReg*** | Jaccard | *0.97 +/- 0.005* | 0.90 +/- 0.003 | 0.92 +/- 0.006 | 0.68 +/- 0.008 |
| ***CalmAn*** | Jaccard | 0.88 +/- 0.006 | 0.90 +/- 0.003 | 0.93 +/- 0.007 | 0.68 +/- 0.007 |

**Supplemental Table 1: F1 and Jaccard statistics for simulated data, Related to Figure 3.** (upper) Average F1 score for each dataset and method. **Bold** entries indicate statistically significant improvement over cellReg. *Italicized* entries indicate statistically significant improvement over CaImAn. *Spatial* indicates SCOUT without temporal similarity metrics. (lower) Average Jaccard similarity accessing the ability of each method in identifying neurons for each dataset. **Bold** entries indicate statistically significant improvement over cellReg. *Italicized* entries indicate statistically significant improvement over CaImAn.
